# The chaperone HSPB1 prepares protein aggregates for resolubilization by HSP70

**DOI:** 10.1038/s41598-021-96518-x

**Published:** 2021-08-24

**Authors:** Conrado C. Gonçalves, Itai Sharon, T. Martin Schmeing, Carlos H. I. Ramos, Jason C. Young

**Affiliations:** 1 Department of Biochemistry, McGill University, 3655 Promenade Sir William Osler, Room 900, Montreal, QC H3G 1Y6 Canada; 2 Department of Biochemistry, McGill University, 3649 Promenade Sir William Osler, Room 457, Montreal, QC H3G 0B1 Canada; 3 Institute of Chemistry, University of Campinas (UNICAMP), Campinas, SP 13083-970 Brazil

**Keywords:** Chaperones, Protein aggregation, Chaperones

## Abstract

In human cells under stress conditions, misfolded polypeptides can form potentially cytotoxic insoluble aggregates. To eliminate aggregates, the HSP70 chaperone machinery extracts and resolubilizes polypeptides for triage to refolding or degradation. Yeast and bacterial chaperones of the small heat-shock protein (sHSP) family can bind substrates at early stages of misfolding, during the aggregation process. The co-aggregated sHSPs then facilitate downstream disaggregation by HSP70. Because it is unknown whether a human sHSP has this activity, we investigated the disaggregation role of human HSPB1. HSPB1 co-aggregated with unfolded protein substrates, firefly luciferase and mammalian lactate dehydrogenase. The co-aggregates formed with HSPB1 were smaller and more regularly shaped than those formed in its absence. Importantly, co-aggregation promoted the efficient disaggregation and refolding of the substrates, led by HSP70. HSPB1 itself was also extracted during disaggregation, and its homo-oligomerization ability was not required. Therefore, we propose that a human sHSP is an integral part of the chaperone network for protein disaggregation.

Cellular proteostasis is essential for proper cell function and survival, but different types of stress and defective physiological conditions, such as mutations or aging, can disturb it by promoting protein misfolding^1–4^. Misfolded proteins can generate insoluble aggregates that accumulate and become cytotoxic^4–6^. Several neurodegenerative disorders including Alzheimer disease, Parkinson disease, and amyotrophic lateral sclerosis (ALS), are caused by the progressive dysfunction and loss of neurons associated with the aggregation and accumulation of amyloids or amorphous aggregates in the cell^3,7–10^.

In order to cope with these aggregates, cells have developed different strategies^11,12^, including compartmentalization into aggresomes followed by autophagy^13–15^, secretion to the extracellular environment^16^ and disaggregation (or resolubilization)^17–20^. Disaggregation performed mainly by molecular chaperones, likely the fastest process, is generally followed by protein refolding or proteasomal degradation^12,21–23^. Previous work has shown that in humans and other metazoans, the chaperone HSP70 together with its co-chaperones DNAJB1 (DJB1), DNAJA2 (DJA2) and HSP110/APG2 are capable of disaggregation^24–28^. The J-domain proteins DJA2 and DJB1 are thought to cooperate to bind the aggregates, recruit HSP70 and stimulate its ATP hydrolysis and substrate polypeptide binding^24,26,28,29^. Also, these experiments suggest that the nucleotide exchange factors (NEFs) HSP110 or its close homolog APG2 are able to support HSP70 disaggregation activity by promoting ADP release and re-binding of ATP, which causes substrate release^24,26,28–30^.

Although, traditionally, protein aggregation has been seen as an uncontrolled process in cells, many cellular factors have been identified as responsible for modulating the aggregation process^11,31,32^. Members of the small heat shock protein (sHSP) family are ATP-independent chaperones, present in all organisms from bacteria to humans, and able to bind to early-unfolding intermediates of substrates, preventing their uncontrolled aggregation^33–35^. In a separate function, sHSPs can be trapped inside protein aggregates, to facilitate downstream disaggregation. Yeast and bacterial sHSPs, Hsp26^24,26^ and IbpA/IbpB^36–38^ respectively, have been reported to co-aggregate with substrates, changing aggregate size, shape and composition, and increasing the efficiency of disaggregation by the combined HSP70 and HSP100 machinery^20,22,23,26,38–41^. The HSP100 disaggregases are absent in metazoans, so it is possible that sHSPs have a conserved role in co-aggregating with substrates to promote efficient disaggregation by the metazoan HSP70 system alone. However, this has not yet been demonstrated.

The disaggregation requirement for co-aggregating sHSP may depend on the properties of the aggregates. It was reported that aggregates of chemically denatured luciferase, or amyloid fibrils of α-synuclein and tau, could be disaggregated by human HSP70 without any co-aggregating sHSP^24,25,27–29^. However, disaggregation of amorphous aggregates of heat denatured luciferase, α-glucosidase or malate dehydrogenase by human HSP70 and transient DJB1-DJA2 complexes required co-aggregation of yeast Hsp26^24,26^. Humans express ten different sHSP (HSPB1-10) and it is likely that some of them might perform a similar role.

Structurally, sHSP are characterized by their low molecular mass (12–43 kDa) and by the presence of a conserved α-crystallin domain (ACD) flanked by flexible N- and C-terminal regions (NTR and CTR)^42–47^. The ACD is relatively conserved and participates in the formation of homo- and hetero-dimerization observed in most proteins of this family^42,43,48,49^. In contrast, NTR and CTR are variable in length and sequence and are involved in substrate binding and regulation of oligomeric state and activity^50–53^, which confers high diversity for the sHSPs^54^. As a consequence of interactions between ACD with NTR and CTR, some sHSPs form large dynamic heterogeneous oligomers^55–57^ and both function and oligomeric state are regulated by post-translational modifications^55,58,59^.

Due to the low sequence homology between sHSP across species, it is difficult to predict which human sHSP homolog of Hsp26 or IbpA/IbpB might be involved in disaggregation. Mouse Hspb1 formed large oligomeric complexes with denatured citrate synthase, which could then be reactivated by Hsp70^60^. However, in the absence of co-chaperones it is unclear whether actual disaggregation was being observed, as opposed to refolding of soluble substrate that had a high rate of spontaneous dissociation from complexes. Human HSPB5 was reported to promote disaggregation of α-synuclein by HSC70 (the constitutive form of HSP70) and co-chaperones, but was not involved in the co-aggregation step, since it was added to pre-formed fibers; furthermore, it resulted in depolymerization over 10 days^61^, much slower than the disaggregation in 1–3 h typically observed with HSC70 or HSP70^24,26–28^. Nevertheless, human HSPB1 may still be an obvious candidate. HSPB1 is ubiquitously expressed in different tissues, especially under stress conditions^62–64^. Also, the ability of HSPB1 to bind to a wide spectrum of substrates has been extensively described^50,64–67^, avoiding the formation and elongation of amyloid fibrils, preventing aggregation of misfolded polypeptides and indirectly promoting their refolding or degradation. In fact, indirect evidence in cells suggested a role of HSPB1 in the reactivation of aggregated substrate^68,69^. In these experiments, human HSPB1 expressed in heat shocked mouse fibroblasts was shown to colocalize with inclusions of luciferase and promote its refolding^69^. Moreover, genetics studies have directly associated HSPB1 mutants with neuropathies, like Charcot-Marie-Tooth disease^70–75^. Some mutations contribute to the formation of larger oligomers than normal, leading to the aggregation of HSPB1 itself and other protein substrates^75,76^. Recent studies have also characterized in detail the interactions between HSPB1 and the intrinsically disordered microtubule-associated protein tau, the deposition of which causes Alzheimer disease and dementia^50,52,77^. Stress-induced phosphorylation of three serine sites (Ser15, Ser78 and Ser82) is essential to activate HSPB1 chaperone function by inducing dissociation of oligomers into dimers and enabling substrate binding^77^. This mechanism agrees with the recently noted chaperone code of combinatorial, regulatory post-translational modifications found on many chaperones ^110,^^111^. Phosphomimetic mutants of HSPB1 have been demonstrated to recapitulate its chaperone function^55,58,59,69,78,79^.

Here we investigate the hypothesis that HSPB1 can co-aggregate with denatured protein substrates, to enable the protein disaggregation led by the HSP70 machinery in an entirely human chaperone system. The activated phosphomimetic mutant HSPB1-3D was able to co-aggregate with two model protein substrates, firefly luciferase and mammalian lactate dehydrogenase (LDH). HSPB1-3D caused smaller aggregates to form than in its absence, although substrates retained the intermolecular contacts typical of aggregates. Also, the HSP70 machinery was only able to disaggregate and refold the substrates when aggregates were formed with HSPB1-3D. Further mutation of HSPB1-3D suggested that self-oligomerization of HSPB1 is not absolutely required for substrate disaggregation. We therefore propose a mechanism for disaggregation in humans in which HSPB1 has a key co-aggregation function.

## Results

### HSPB1-3D co-aggregates with substrate to regulate aggregate size and morphology

**Figure 1:**
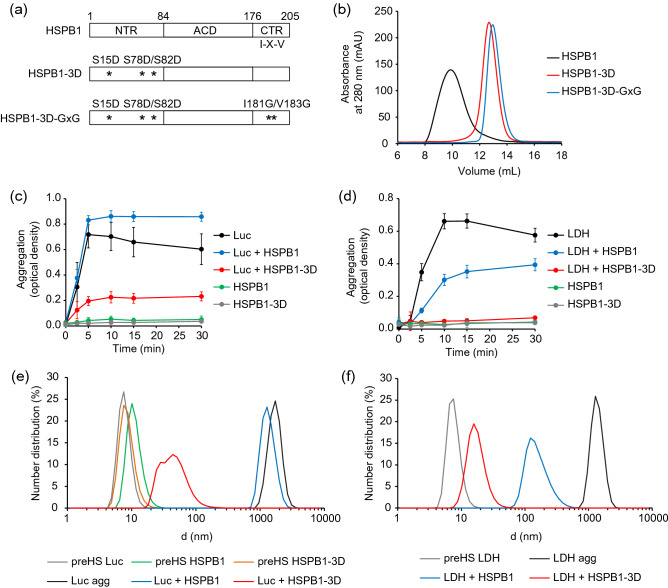
HSPB1-3D co-aggregates with substrate forming smaller species. (**a**) Domain organization of HSPB1 constructs. Full-length HSPB1 is composed of the α-crystallin domain (ACD) flanked by the N- and C-terminal regions (NTR and CTR). Amino acid numbers, and the IXI/V motif are labeled. Three phosphorylatable serine residues found in the NTR (Ser15, Ser78 and Ser82) are highlighted. The phosphomimetic mutant, HSPB1-3D, was obtained by the substitution of those serines to aspartates. The IXI/V motif (^181^IPV^183^) of the HSPB1-3D mutant was further mutated into ^181^GxG^183^, generating the dimeric HSPB1-3D-GxG mutant. (**b**) Oligomerization state of 20 µM HSPB1 and its mutants was assessed by SEC analysis. Aggregation of 2 µM (**c**) luciferase or (**d**) LDH under heat shock (45 °C or 55 °C, respectively) in the absence or presence of 20 µM HSPB1 or HSPB1-3D. The substrate aggregation was assessed by measurement of optical density at 320 nm. Optical densities of HSPB1 and HSPB1-3D alone under heat shock were measured as controls. Error bars show standard deviations, n ≥ 3. (**e**, **f**) The size distributions of HSPB1 and HSPB1-3D, as well as native pre-heat shock (preHS) and aggregated (agg) (**e**) luciferase and (**f**) LDH were estimated by dynamic light scattering (DLS).

Initially, we focused on characterizing the interaction of HSPB1 with aggregating proteins. Phosphorylation of specific serine residues in the NTR is known to regulate its oligomeric state and activity, and the HSPB1-3D phosphomimetic mutant that substitutes these serines for aspartates (Fig. 1a) reconstitutes its chaperone activity^55,58,59^. Size exclusion chromatography (SEC) analysis of HSPB1 and the 3D mutant showed, as expected^58,80,81^, that HSPB1 is a large heterogenous oligomer that eluted as a broad peak close to the void of the column, while HSPB1-3D was eluted later in the column as a narrower peak, indicating that the mutation disassembles the oligomers into smaller oligomers and possibly dimers (Fig. 1b).

We verified the abilities of HSPB1 and HSPB1-3D to bind substrates that were in the process of forming aggregates. Assays for the prevention of aggregation by chaperones were conducted using firefly luciferase, an established model for Hsp70-dependent refolding^82^, and lactate dehydrogenase (LDH), an important mammalian metabolic enzyme. The formation of aggregates over time under heat shock was monitored by light scattering, measured by the optical density of solutions containing the substrates in the absence or presence of HSPB1. Luciferase aggregated substantially after 5 min at 45 ℃ and a similar high optical density was observed upon incubation with HSPB1 (Fig. 1c). In contrast, aggregates formed with HSPB1-3D showed 70% less optical density (Fig. 1c), suggesting that this mutant binds luciferase and forms either fewer or smaller aggregates compared to the aggregation of luciferase alone.

The aggregation of LDH in the absence of sHSP occurred after 10 min at 55 ℃ (Fig. 1d). Differently from luciferase, LDH aggregates formed with HSPB1 showed a decrease of 45% of the optical density, indicating that HSPB1 in its oligomeric form was able to interact with LDH. HSPB1-3D was much more efficient as a chaperone, since LDH aggregates had ~ 90% less optical density when formed in its presence. Nonetheless, some aggregates still formed under these conditions (Fig. 1d).

We performed DLS measurements to obtain further information on the sizes of HSPB1 constructs and the aggregates (Fig. 1e and f). The sizes of native luciferase and HSPB1-3D were distributed in single narrow peaks in which estimated average diameters were 7.9 and 8.7 nm, respectively (Fig. 1e). Consistently with a larger oligomer, the size distribution of unmodified HSPB1 had a diameter of 11.3 nm. Aggregated luciferase alone or with HSPB1 had diameters corresponding to very large particles (1725 and 1355 nm, respectively, Fig. 1e). Notably, all measurements were determined from monodisperse number distribution curves, except for the luciferase aggregates formed in the presence of HSPB1-3D, which showed a broad polydisperse curve with sizes distributed in two partially overlapping peaks with estimated diameters of 27.5 and 56.4 nm (Fig. 1e). Similarly, native LDH was around 7.7 nm on average and aggregates of LDH alone were around 1383 nm (Fig. 1f). In the presence of HSPB1, the average diameter of LDH aggregates was 163.4 nm, and aggregates were much smaller at 17.7 nm when formed with HSPB1-3D (Fig. 1f). Thus, both luciferase and LDH appear to form heterogeneous aggregates together with HSPB1-3D, or the sHSP may be said to co-aggregate with the substrates.

Conceptually, an aggregate consists of at least two molecules of unfolded polypeptides that contact each other through their exposed hydrophobic portions^4^. In order to confirm that HSPB1-3D bound to aggregated polypeptides, instead of merely preventing their aggregation by binding to monomeric unfolded polypeptides, we designed a cross-linking assay. The substrates were heat shocked to form aggregates in the absence or presence of HSPB1-3D, or incubated at 4 ℃ as control, then submitted to cross-linking reactions with different concentrations of the amine-reactive crosslinker DSS and analyzed by immunoblot. We hypothesized that if aggregates were formed after heat shock, substrate molecules would be close enough to allow the cross-linking reaction between them (i.e. intermolecular luciferase-luciferase or LDH-LDH contacts). Indeed, high molecular mass bands corresponding to cross-linked substrate were only detected in the immunoblots in the samples submitted to heat shock (Supplemental Fig. S1a and b). Moreover, cross-linked substrates were detected both in the absence and the presence of HSPB1-3D, indicating that the contacts between substrate molecules still formed when bound by the chaperone. The data also suggested a difference in the size of the aggregates of LDH alone or with HSPB1-3D, especially at higher concentrations of DSS (40 and 50 µM), in which aggregates of cross-linked LDH alone were too large to be resolved in the SDS-PAGE, while they were detected when the chaperone was present (Supplemental Fig. S1b). Therefore, the actived HSPB1 in fact participates in the aggregation with the substrate, which we term its co-aggregation function.

**Figure 2:**
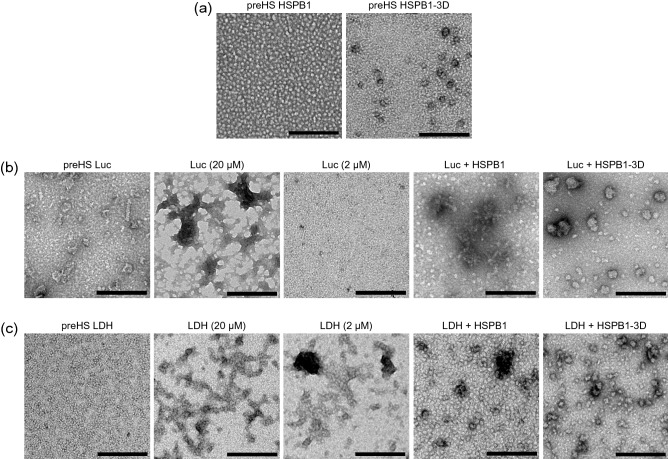
HSPB1-3D regulates the size and morphology of aggregates. (**a**–**c**) Representative negative stained electron micrographs of (**a**) native HSPB1 and HSPB1-3D; and of (**b**) luciferase or (**c**) LDH before and after incubation for 15 min at 45 °C or 55 °C, respectively. The aggregates in the presence of 20 µM HSPB1 or HSPB1-3D were prepared with 2 µM luciferase or LDH. Luciferase alone could not be detected at 2 µM, so preHS and substrate alone samples were prepared also using 20 µM luciferase or LDH. All scale bars represent 200 nm.

Negative stain electron microscopy (EM) analysis of the HSPB1 constructs and the aggregates provided additional information about their size and morphology. Micrographs of HSPB1-3D revealed a population composed of small globular particles, which might correspond to dimers, and larger particles, possibly oligomers still assembled (Fig. 2a). HSPB1 showed a more homogenous population of globular particles, similar in size to the large particles observed in HSPB1-3D samples, consistent with them being assembled oligomers and with previous work^83^ (Fig. 2a). Native luciferase was observed mostly as small globular particles, but we also detected larger particles with irregular shapes, some more globular and some more tubular (Fig. 2b). Because of the low stability of luciferase^84,85^, we interpreted these larger particles as aggregates already formed during its preparation. The native LDH population was homogenous and composed of small globular particles (Fig. 2c). When subjected to heat shock, luciferase and LDH aggregates were observed as a continuous mass of amorphous particles (Fig. 2b and c; lower concentrations of luciferase aggregates were not detected on grids). In contrast, when the substrates were aggregated in the presence of HSPB1-3D, globular aggregates were obtained. The population of luciferase aggregates included particles of different sizes, while LDH aggregates were more homogenous in size (Fig. 2b and c), which is in agreement with our DLS data (Fig. 1e and f). These aggregates are larger than native proteins but much smaller than the aggregates of substrates alone. Moreover, both luciferase and LDH aggregates with HSPB1-3D have a similar globular shape, very different from the amorphous mass of aggregates in the absence of chaperone. Thus, HSPB1-3D may regulate the size and morphology of aggregates during their formation. In the samples where substrates were heat shocked with HSPB1, small particles, corresponding to HSPB1, are the majority in the population, but some very large and sparse particles were also observed, probably corresponding to aggregated substrates (Fig. 2b and c).

### HSPB1-3D is required for aggregate resolubilization

**Figure 3:**
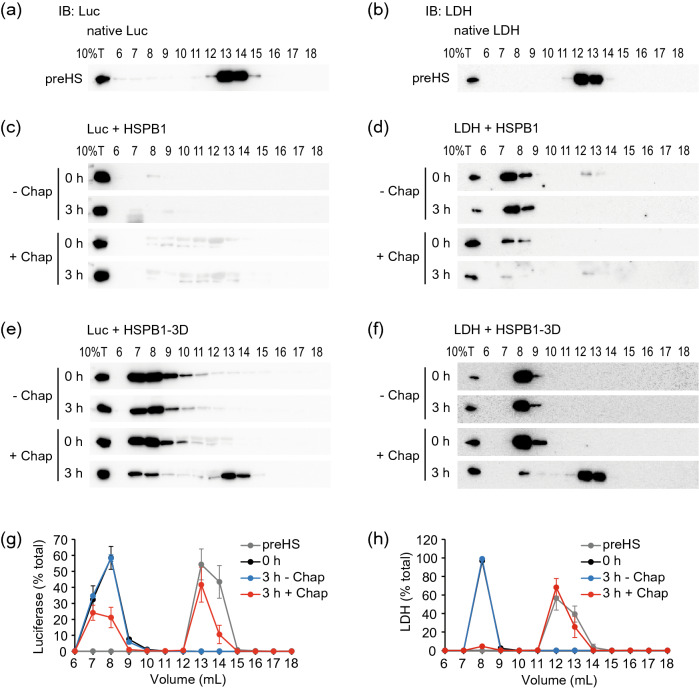
HSPB1-3D is required for aggregate resolubilization. (**a**, **b**) SEC analysis of native (**a**) luciferase and (**b**) LDH. After chromatography, the elution patterns of proteins were detected by immunoblot (IB). For comparison, 10% of total protein (10%T) before chromatography was also detected. (**c**–**f**) Disaggregation of aggregates composed of (**c**) luciferase and HSPB1, (**d**) LDH and HSPB1, (**e**) luciferase and HSPB1-3D, or (**f**) LDH and HSPB1-3D. The aggregates were formed at 45 ℃ or 55 ℃ for 15 min and transferred to disaggregation reactions containing buffer (− Chap) or the chaperone disaggregation machinery (+ Chap) and ATP. Aggregate resolubilization at the start (0 h) and end (3 h) of the disaggregation reactions was analyzed by SEC and immunoblot (IB). (**g**, **h**) Quantification of (**g**) luciferase or (**h**) LDH aggregates with HSPB1-3D from e and f in eluted fractions are shown as percentage of the total amount of immunoblot signal. Error bars show standard deviations, n ≥ 3.

Next, the disaggregation machinery composed of HSP70, DNAJ proteins and HSP110^24,26^ was used to assess the resolubilization of the aggregates formed with HSPB1 and HSPB1-3D. After heat shock at 45 °C or 55 °C, respectively, luciferase or LDH aggregates were transferred to a mix of the chaperones and ATP at 30 °C and analyzed at the start (0 h) and end (3 h) of the disaggregation reactions by SEC and immunoblot (Fig. 3). As controls, native luciferase and LDH were eluted in low molecular size fractions (13–14 mL, and 12–13 mL, respectively, Fig. 3a and b), consistent with native monomers (luciferase) and tetramers (LDH). Their elution patterns were used as a comparison to observe disaggregation. For luciferase aggregates formed with HSPB1, most of the input luciferase was not recovered after SEC, most likely because the large aggregates were trapped in the column (Fig. 3c). LDH aggregates formed with HSPB1 were eluted in the void volume (7–8 mL, Fig. 3d), as well as luciferase and LDH aggregates formed in the presence of HSPB1-3D, indicating their large molecular size (≥ 1 MDa, Fig. 3e and f). The fact that luciferase aggregates with HSPB1-3D were detected in the column, while the ones with HSPB1 were not, is consistent with our above data showing that HSPB1-3D co-aggregates are smaller than aggregates of substrate alone and that HSPB1 does not bind luciferase well (Fig. 1c and e). After the disaggregation reactions containing HSPB1-3D, ~ 55% of luciferase (Fig. 3e and g) and ~ 95% of LDH (Fig. 3f and h) moved from the aggregate fractions back to the low molecular size fractions. In the negative control reactions without the chaperones, both luciferase and LDH remained in high molecular size fractions (Fig. 3c–h). These results indicate that HSP70 and the co-chaperones partially or almost completely extracted polypeptides from the aggregates containing HSPB1-3D back to their original soluble states, luciferase monomers and LDH tetramers.

### HSPB1-3D changes oligomeric state during disaggregation

**Figure 4:**
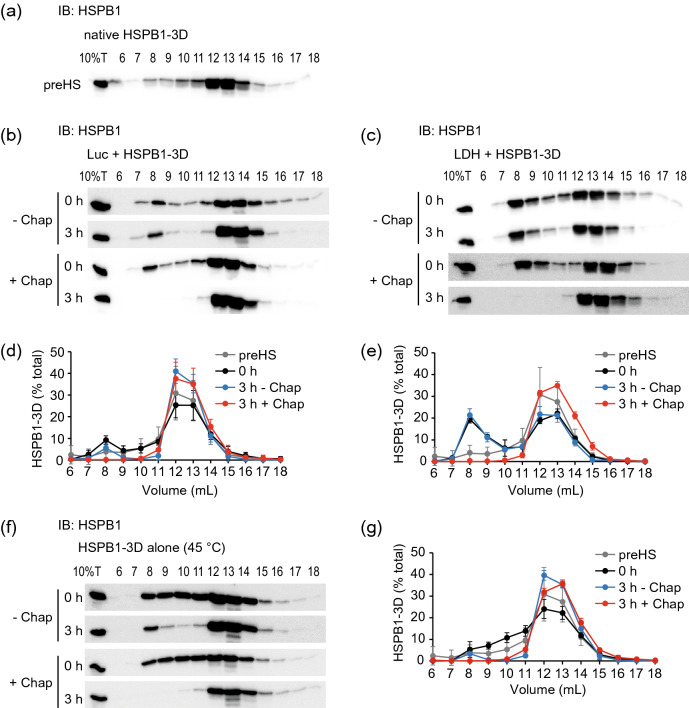
HSPB1-3D changes oligomeric state during disaggregation. (**a**–**c**) The elution patterns of HSPB1-3D (**a**) before the heat shock or in the disaggregation reactions with (**b**) luciferase or (**c**) LDH as in Fig. 3c–f, were assessed by SEC and immunoblot (IB). (**d**, **e**) Quantification of HSPB1-3D in the reactions with (**d**) luciferase and (**e**) LDH from B and C are shown as percentage of the total amount of immunoblot signal. (**f**) HSPB1-3D in the absence of substrates was submitted to heat shock at 45 ℃, transferred to reaction buffer with (+ Chap) or without (− Chap) chaperones and analyzed by SEC and immunoblot. (**g**) Quantification of HSPB1-3D elution pattern in f. Error bars show standard deviations, n ≥ 3.

We also monitored HSPB1 and HSPB1-3D elution patterns during the disaggregation reactions. As shown in Fig. 1b, native pre-heat shock HSPB1 was eluted on SEC in a broad peak close to the void volume, as a large polydisperse oligomer, observed in immunoblots of the fractions (8–13 mL, Supplemental Fig. S2a). Few changes were seen in the HSPB1 elution pattern due to heat shock (Supplemental Fig. S3a and b) or presence of substrate (Supplemental Fig. S2b-d), except for a slight shift of the peak in the direction of the void volume immediately after heat shock. In contrast, the great majority (~ 90%) of native HSPB1-3D was in the low molecular size fractions although a small amount eluted throughout the column (Fig. 4a). Immediately after the heat shock (0 h disaggregation) with luciferase or LDH, ~ 20% and 40%, respectively, of the total amount of HSPB1-3D was found in high molecular size fractions (7–9 mL) compared to the 10% of the native preHS sample (Fig. 4b–e). This shift to high molecular size is consistent with a portion of HSPB1-3D co-aggregating with the substrate during heat shock. Also, after the disaggregation reaction (3 h), all the high molecular size HSPB1-3D moved back to the low molecular size fractions, suggesting that HSP70 and co-chaperones helped to extract HSPB1-3D from the co-aggregates with the substrate (Fig. 4b–e). Interestingly, in the absence of chaperones, part of HSPB1-3D remained as a high molecular size species, probably trapped in co-aggregates with the substrate (Fig. 4b–e). Another possibility is that HSPB1-3D in the high molecular size fractions formed oligomeric interactions with itself as well as with substrate. If so, HSP70 and the disaggregation machinery may also help to disassemble such oligomers.

To confirm if the shift in HSPB-3D molecular size was only due to the binding with substrate, the same experiment was performed in the absence of luciferase or LDH. We observed that heat shock at 45 °C (Fig. 4f and g) and 55 ℃ (Supplemental Fig. S3c and d) was enough to cause ~ 25% of HSPB1-3D to shift its elution to the high molecular size fractions, consistent with homo-oligomer formation. The addition of HSP70 and the disaggregation machinery brought all HSPB1-3D back to the low molecular size fractions, whereas in the absence of chaperones, ~ 5% remained as larger species after 3 h (Fig. 4f and g, Supplemental Fig. S3c and d). These data suggest that changes in the temperature and the HSP70 machinery might play a role in regulating the HSPB1 oligomeric state. Moreover, the high molecular weight forms of HSPB1-3D observed in the presence of substrate might be a mixture of chaperone oligomers and co-aggregated substrate.

### HSPB1-3D-dependent disaggregation results in refolding

**Figure 5:**
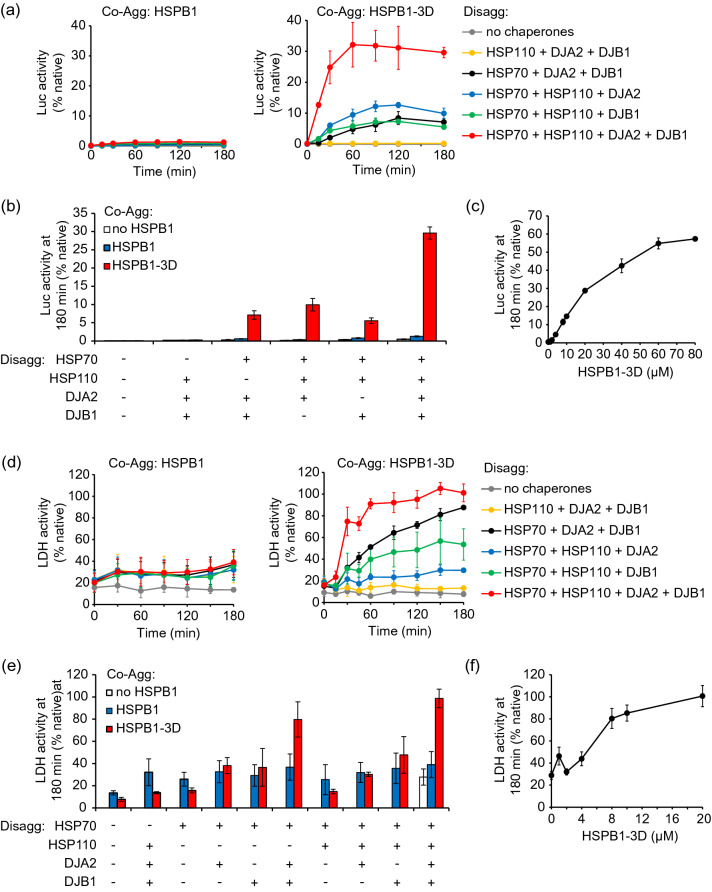
HSPB1-3D-dependent disaggregation results in refolding. Disaggregation reactions with heat shocked (**a**–**c**) luciferase or (**d**-**f**) LDH aggregates formed in the absence or presence of HSPB1 or HSPB1-3D were performed as described above. Substrate enzymatic activities were monitored over time in reactions containing the indicated chaperone combinations. Kinetics of (**a**) luciferase and (**d**) LDH reactivation from aggregates with HSPB1 or HSPB1-3D are reported relative to native substrate activity. (**b**) Luciferase and (**e**) LDH enzymatic activities as in a or d, after 3 h of disaggregation reaction with different combinations of chaperones. (**c**) Luciferase or (**f**) LDH enzymatic activities after 3 h disaggregation reaction. Error bars show standard deviations, n ≥ 3.

We performed disaggregation assays in which recovery of substrate enzymatic activity was monitored to assess not only the extraction of polypeptides from aggregates, but also their refolding back to the native functional state. Native substrate activity before the heat shock (preHS) was measured and set as maximum. Also, different combinations of chaperones were used to determine the minimum chaperone requirement allowing efficient disaggregation and refolding of the substrates. Predictably, when luciferase aggregates were prepared in the absence of sHSP or in the presence of HSPB1 no reactivation of luciferase was observed, regardless of the combination of chaperones tested (Fig. 5a and b). However, substrates in aggregates formed with HSPB1-3D (in a ratio 1:10, luciferase:HSPB1-3D) recovered up to 30% of their activity when all the components of the HSP70 disaggregation machinery were present, i.e., HSP70, HSP110, DJA2 and DJB1. If HSP70 was absent no reactivation was obtained, and the absence of each of the co-chaperones individually reduced the reactivation to 10% or less (Fig. 5a and b). Increasing the concentration of HSPB1-3D during the aggregation step caused an increase in luciferase reactivation of up to 60% in ratios of 1:30 or 1:40 (Fig. 5c). However, a similar increase in monomeric luciferase at higher ratios of HSPB1-3D was not observed in SEC assays detecting aggregate resolubilization directly. In these assays, around 60% of luciferase was disaggregated either in the presence of 20 µM or 80 µM HSPB1-3D (i.e. 1:10 and 1:40 ratios, respectively, Fig. 3e and g, Supplemental Fig. S4a and b). This result suggests that the ratio 1:10 is sufficient for the formation of smaller co-aggregates of chaperone with the portion of substrates that can be disaggregated, but higher amounts of HSPB1-3D might help somehow during the luciferase refolding process. Slightly more HSPB1-3D shifted to higher molecular size fractions at 80 µM than at 20 µM, but most of it returned to the low molecular size population after the disaggregation reaction (Supplemental Fig. S4a, c and d), suggesting that the mechanism of the chaperone in co-aggregation and disaggregation was unchanged.

To distinguish the requirements for disaggregation from those for refolding, luciferase was denatured by guanidinium-Cl (GdmCl) to be entirely monomeric and unfolded, and its refolding was analyzed. As previously observed^82,86^, luciferase refolding by HSP70 and DJA2 was efficient by 60 min (Supplemental Fig. S5a and b). HSP70 and DJB1 had poor refolding activity, the same as with HSP70 alone or HSP70 and HSP110. In fact, the addition of HSP110 in these conditions had an inhibitory effect on HSP70 and DJA2 refolding activity, whereas DJB1 did not interfere (Supplemental Fig. S5a and b). These data suggest that although all the components together are important for the resolubilization of luciferase aggregates, HSP70 and DJA2 as previously established^86^ are sufficient to refold the substrate to its native conformation.

The recovery of activity after disaggregation was also investigated for LDH aggregates. The results showed that reactivation of LDH from aggregates formed without sHSP or with HSPB1 reached 30% and 40%, respectively, compared to native activity (Fig. 5d and e). Much more efficient recovery was obtained after reactions with aggregates prepared with HSPB1-3D. Maximum reactivation (98%) was observed in the reactions containing all the chaperone components of the disaggregation machinery (Fig. 5d and e). As observed for luciferase, HSP70 was an essential element for disaggregation since very low LDH reactivation was achieved in its absence, comparable to the absence of any chaperone (10%). When DJA2 or DJB1 were absent individually, reactivation was reduced to 50% and 30%, respectively; and if both were removed from the reactions, only 15% of the activity was rescued, highlighting the importance of the DnaJ proteins for aggregate resolubilization. Differently from luciferase, however, the absence of HSP110 only reduced final LDH reactivation to 80%. In fact, HSP110 was more important to enhance the velocity of reactivation coupled to disaggregation in the first 30 min of reaction, when 70% of the activity was recovered in the presence of HSP110 compared to 30% in its absence (Fig. 5d). Titration of HSPB1-3D demonstrated that the ratio of 1:10 (LDH:HSPB1-3D) was sufficient to reactivate LDH completely (Fig. 5f). Smaller ratios, such as 1:5 or 1:4, were also efficient, achieving up to ~ 85% reactivation (Fig. 5f).

The refolding of GdmCl-denatured LDH showed that this substrate required the support of chaperones to reach its functional conformation, since only low activity was observed in reactions without chaperones (Supplemental Fig. S5c and d). However, unlike for luciferase, LDH refolding did not have a specific requirement for HSP70 and DJA2. Substantial LDH refolding was obtained under all the combinations containing chaperones, including in the absence of HSP70 (Supplemental Fig. S5c and d). This behaviour might indicate that chaperones are important to prevent unfolded LDH from aggregating, but that the refolding process is spontaneous (chaperone-independent), which could be one explanation why the levels of LDH reactivation after disaggregation were much higher than luciferase. Moreover, the reactivation differences observed between combinations of chaperones in disaggregation reactions (Fig. 5d and e) might be attributed solely to disaggregation efficiency.

### Dynamics of HSPB1 oligomeric state

In order to understand if the change observed in HSPB1-3D oligomeric state plays a role during disaggregation of the substrate, we used the mutant HSPB1-3D-GxG (I181G/V183G). This mutation in the CTR IXI/V motif (^181^IPV^183^) of HSPB1 (Fig. 1a) disrupts the interactions between ACD and CTR and, combined with the 3D mutation, results in a well characterized monodisperse HSPB1 dimer, unable to oligomerize^50,52,87^. SEC analysis of this mutant showed a narrow peak eluted slightly after HSPB1-3D (Fig. S1b), consistent with the size of a homogeneous dimer (~ 50 KDa).

We hypothesized that if the change in the oligomeric state is important, then HSPB1-3D-GxG would not be able to assist the HSP70 machinery in the disaggregation of substrate. First, we addressed its ability to bind and co-aggregate with denatured luciferase and LDH. Surprisingly, HSPB1-3D-GxG did not decrease the optical density of luciferase aggregates formed during heat shock (Supplemental Fig. S6a). In contrast, its presence during the heat shock of LDH resulted in 95% less optical density than with the aggregating substrate alone (Supplemental Fig. S6b). The size distribution of the aggregates measured by DLS corroborated these results, since the estimated diameter of luciferase aggregates with HSPB1-3D-GxG was 240.7 nm (Supplemental Fig. S6c) in agreement with large aggregates, while LDH aggregates were 22 nm (Supplemental Fig. S6d), similarly to the ones formed with HSPB1-3D (Fig. 1f). The cross-linking assay confirmed that LDH formed intermolecular contacts with itself in the presence of HSPB1-3D-GxG, consistent with aggregate formation (Supplemental Fig. S1c). However, unlike HSPB1-3D, no cross-linked HSPB1-3D-GxG species larger than a dimer was detected (Supplemental Fig. S1b and c), consistent with the inability to form oligomers. These results suggest that HSPB1-3D-GxG effectively changed the aggregation properties of LDH but not luciferase, by co-aggregating as dimers.

**Figure 6:**
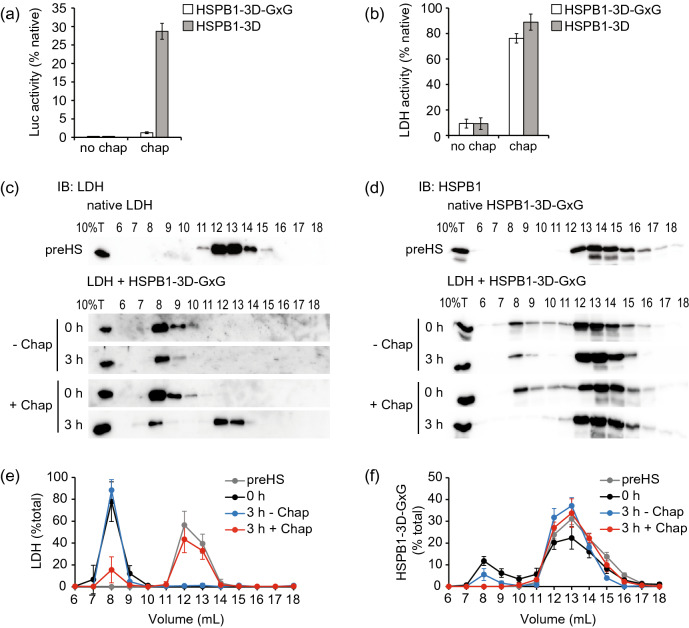
HSPB1-3D-GxG assists disaggregation of LDH but not luciferase. (**a**, **b**) Disaggregation of (**a**) luciferase and (**b**) LDH aggregates prepared with or without HSPB1-3D-GxG was assessed through the measurement of substrate enzymatic activity recovery as in Fig. 5. Aggregates prepared with HSPB1-3D were used as positive controls and reactions in the absence of chaperones were used as negative controls. Native substrate activity was set as 100% activity. (**c**, **d**) The effect of HSPB1-3D-GxG in LDH disaggregation. (**c**) LDH and (**d**) HSPB1-3D-GxG elution patterns at the beginning (0 h) and end (3 h) of reactions with (+ Chap) or without (− Chap) chaperones were determined by SEC and immunoblot (IB) as in Fig. 3. (**e**, **f**) Quantification of (**e**) LDH or (**f**) HSPB1-3D-GxG from C and D in eluted fractions are shown as percentage of the total amount of immunoblot signal. Error bars show standard deviations, n ≥ 3.

Next, we evaluated if the aggregates formed with HSPB1-3D-GxG could be resolubilized by the HSP70 disaggregation machinery. Luciferase in larger aggregates showed no reactivation of its enzymatic activity after disaggregation reactions (Fig. 6a). However, 75% of LDH was reactivated after disaggregation reactions containing HSP70 and co-chaperones, compared to 9% in reactions without them (Fig. 6b). We confirmed the disaggregation of LDH by SEC analysis. 75% of the aggregated LDH at the beginning of the disaggregation reactions was found in the native molecular size fractions after 3 h in the presence of chaperones (Fig. 6c and e), consistent with the activity recovery mentioned above. Interestingly, even though HSPB1-3D-GxG was mainly eluted as a dimer before heat shock, 20% of it moved to the large molecular size fractions during the co-aggregation with LDH (Fig. 6d and f). After the disaggregation reaction, if chaperones were present, LDH returned to the low molecular size fractions and all HSPB1-3D-GxG shifted back to the dimeric size (Fig. 6d and f). In control reactions without chaperones, a portion of HSPB1-3D-GxG remained in the high molecular size fractions along with the substrate (Fig. 6d and f). When HSPB1-3D-GxG was heat shocked in the absence of substrate (Supplemental Fig. S7a and b) or with luciferase (Supplemental Fig. S7c), no shift towards the high molecular size fractions was observed, confirming that the oligomers observed with LDH were co-aggregates with that substrate. Thus, the intrinsic oligomerization activity of the HSPB1 chaperone is not necessary in all cases for its function in disaggregation.

## Discussion

**Figure 7:**
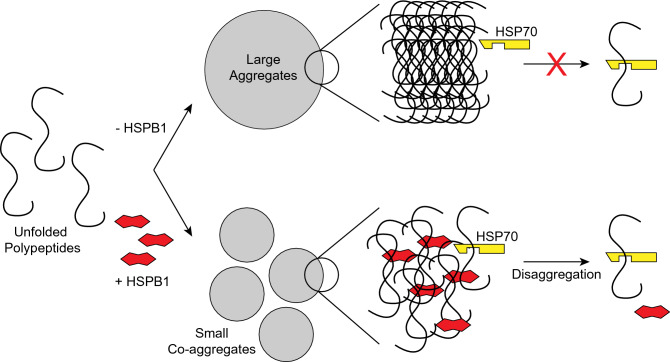
Model for the mechanisms of HSPB1 in aggregate solubilization. Unfolded polypeptides subjected to heat shock in the absence of HSPB1 form large amorphous aggregates that cannot be disaggregated by the HSP70 machinery. In the presence of HSPB1, polypeptides co-aggregate with the sHSP generating small globular aggregates. Under these conditions, the HSP70 disaggregation machinery, formed by HSP70 and co-chaperones (DNAJ proteins and HSP110), extracts substrate and HSPB1 dimers from aggregates.

Taken together, our results support a model of disaggregation in which unfolded polypeptide co-aggregates with activated HSPB1 (Fig. 7). As part of a high molecular mass co-aggregate, HSPB1 changes the properties of the aggregates in a way that facilitates the extraction and refolding of polypeptides. HSP70 and its co-chaperones, the DNAJ proteins and HSP110, extract substrate polypeptides and release dimers of HSPB1 from the aggregates. The substrate may then be refolded, and HSPB1 could be recycled for new rounds of co-aggregation and disaggregation (Fig. 7).

The aggregates formed with HSPB1-3D are substantially smaller than in its absence, as shown by DLS (Fig. 1e and f). It is possible that HSPB1-3D makes some aggregates less dense, or more loosely connected, to allow better disaggregation. The difference in size alone may promote disaggregation by increasing the number of substrate molecules exposed on the surface of aggregates. However, decreasing the size of aggregates is not sufficient for disaggregation activity, as observed for luciferase and HSPB1-3D-GxG (Supplemental Fig. S6c, Fig. 6a, Supplemental Fig. S7c). Our negative staining EM micrographs confirmed that aggregates formed with HSPB1-3D were much smaller and revealed a globular shape compared to the large mass of amorphous aggregates formed with substrate alone or with inactive HSPB1 (Fig. 2b and c). EM analysis of luciferase aggregates with bacterial IbpA^36^ and of citrate synthase aggregates with yeast Hsp26 or mouse Hspb1^22,60,88^ showed particles very similar in size and morphology compared to the ones obtained here. It is likely that aggregate size is a consequence of HSPB1-3D binding to substrate, rather than the size determining the binding of the chaperone. HSPB1-3D would bind hydrophobic sequences within polypeptides while they are still soluble, at the very start of aggregation ^33,34,53^. As co-aggregates form, HSPB1-3D would slow but not stop the growth of aggregate particles.

In fact, the capacity of sHSP to control aggregate formation in an organized manner seems to be evolutionarily conserved^35^ since it has also been described in bacteria^23,36–38^, yeast^34,37^ and plants^23,39^. In accordance with our data, the sHSP-arranged aggregates are efficiently disassembled and refolded by the HSP70 machinery and HSP100 family disaggregases in these organisms. In bacteria, aggregates formed in the presence of IbpA/B were more easily solubilized by DnaK and ClpB (bacterial HSP70 and HSP100, respectively) than in their absence^36–38^. Similarly to what we observed, DnaK had a specific role to bind IbpA/B and release it from the aggregates as the first step in the solubilization reaction^37^. The mechanisms to bind substrates as dimers is also similar between human sHSP and other organisms. IbpA and IbpB from bacteria are constitutively active dimers and cannot oligomerize^37^, whereas human sHSP, as well as Hsp26 and Hsp42 from yeast^89,90^, can form large oligomers but dissociate into smaller species to become active chaperones. Our data are in line with previous studies^51,58,59^ in suggesting that dissociation of HSPB1 into smaller species is crucial for its chaperone activity, including co-aggregation.

The presence of the disaggregation mechanism in all organisms indicates its importance for proteostasis. Polypeptide resolubilization is likely to be a highly efficient mechanism for cells to eliminate potentially toxic aggregates in response to stress, by supporting refolding or clearance by the ubiquitin–proteasome system. The co-aggregation of HSPB1 or possibly other sHSPs could promote disaggregation on fast, biologically relevant time scales. According to our data, refolding is completed in 60 min (Supplemental Fig. S5a and c), while substrate resolubilization and refolding happened after 120 min (Fig. 5a and d), suggesting that disaggregation is comparably fast. Experiments in cells support this hypothesis. Heat shocked human cells expressing luciferase were able to resolubilize and reactivate luciferase inclusions or aggregates after 2 h of recovery^82,91^. Fibrils of α-synuclein from *C. elegans* muscle cells were disassembled after 4 h incubation with HSC70, DJB1 and APG2^25^. Also, HeLa cytosol disaggregated luciferase and GFP aggregates after incubation of 4 h or more^92^. Alternatively, in human cells, large aggregates can be cleared by autophagy without having to be dissociated into individual polypeptides^14,15,93^. The relative importance of aggregate solubilization remains to be determined, but we propose that it has advantages over autophagy for the cell. Disaggregation appears to be comparatively fast, and if the substrates are not refolded afterward, degradation by proteasomes is also expected to be fast. Solubilization followed by degradation may then be a more efficient use of cellular resources.

As previously established^24–26^, the HSP70 chaperones are the main motors for substrate disaggregation; and HSP70, DJB1 and HSP110 are the minimal requirements for efficient disaggregation of amorphous and amyloid aggregates^24–28^. Interestingly, sHSP co-aggregation appears to be most important for solubilization of amorphous aggregates, but not of amyloid fibrils^24–29^. In accordance, we obtained the most efficient substrate disaggregation in the presence of HSP70, DJA2, DJB1 and HSP110. According to the ATP-dependent entropic-pulling model, HSP70 molecules recruited by co-chaperones (DNAJ proteins) bind to hydrophobic peptide segments exposed at the aggregate surface. When bound adjacent to a large aggregate, the freedom of motion of HSP70 is restricted, so any movement away from the aggregate increases motional freedom and entropy, and causes an entropic pull that extracts the polypeptide^94,95^. As a consequence, crowding of HSP70 molecules on the aggregates could increase disaggregation efficiency^28^. The role of HSPB1 in regulating substrate aggregation fits well into this model. In comparison with the extremely large aggregates of substrate alone, as observed by EM (Fig. 2b and c), the smaller aggregates formed with HSPB1 offer a larger surface area and possibly more exposed peptide segments where HSP110, DNAJ proteins and HSP70 can bind. Also, the smaller size could allow more crowding of the HSP70 machinery, leading to a more efficient disaggregation.

The participation of HSPB1 for efficient resolubilization and refolding of protein aggregates is in line with many studies reporting its role in protein-misfolding diseases^96,97^. ALS mice models expressing different SOD1 mutations have shown upregulation of HSPB1 expression in the spinal cords^98^. Moreover, HSPB1 overexpression was beneficial against the damaging effects of SOD1 mutants in ALS neuronal cell models^99,100^. Also, in vitro studies have observed HSPB1 binding to aggregate-prone proteins, such as tau^50,52^, α-synuclein^67,101,102^ and SOD1 mutants^103^. Mutations on HSPB1 could affect its co-aggregation function and be responsible for serious consequences on many cellular processes. For example, in the axonal form of Charcot-Marie-Tooth disease, HSPB1 mutants are associated to cause cell death due to cytoskeletal damage^72,75,104^. Specifically, the S135F HSPB1 mutant has been shown to aggregate and disrupt the neurofilament network, which leads to progressive degeneration and loss of viability of motor neurons, characteristic of this disease^72^.

The process of disaggregation in cells might be more complicated if we consider that other human sHSP could be involved. Similarly to HSPB1, expression of HSPB5 and HSPB8 was upregulated in muscle and neurons cells of transgenic SOD1 mutant mice^98^ and their induced overexpression was able to reduce SOD1 mutant aggregation in vitro^103,105^. In fact, as demonstrated by screening studies with different human sHSP^64,83^, binding affinity and chaperone activity vary according to the substrate, so presumably sHSPs other than HSPB1 might also have co-aggregation activity, increasing the spectrum of substrates under the protection against irreversible aggregation.

HSPB8 also stands out as together with HSP70, the nucleotide exchange factor BAG3 and the E3 ubiquitin ligase CHIP/STUB1, it forms the CASA (chaperone assisted selective autophagy) complex responsible for ubiquitinating misfolded substrates and initiating the formation of autophagosomes for their degradation^97,106^. Moreover, HSPB8, BAG3 and HSP70 are important for the protection of stress granules (SG)^107^. SGs are complexes of RNA-binding proteins that sequester mRNA under cellular stress^108^ and allow translation restoration when stress attenuates. However, aberrant misfolded polypeptides can also accumulate in SGs and convert them into insoluble aggregates. HSPB8 also accumulated in the SGs prevents irreversible protein aggregation inside them, and then recruits HSP70 and BAG3 to promote polypeptide extraction, maintaining the function of SGs^107^. The modulation of SG properties by HSPB8 may parallel the function of HSPB1 to regulate properties of amorphous protein aggregates that we propose here. However, HSPB1 has only a mild effect in the maintenance of stress granules^107^, suggesting that different types of aggregates, or different disaggregation mechanisms, might require the activity of specific sHSPs.

## Experimental procedures

### Protein synthesis and purification

Unless otherwise stated, all chemical reagents were from BioShop Canada or Sigma. Human HSP70 and DJA2 inserted in pPROEX HTa were expressed and purified as published^82,86^. His-SUMO-tagged human HSP110 in pET28a was a gift from Prof. Rina Rosenzweig (Weizmann Institute of Science). Human Frt-V5-HSPB1 (63102) and pcDNA5/FRT/TO DNAJB1 (19468) were from AddGene. Their coding sequences were amplified by PCR and inserted into pPROEX HTa and pET28a, respectively. The S15D, S78D and S82D mutations were inserted in HSPB1 to make HSPB1-3D by site-directed mutagenesis. The mutations I181G and V183G were inserted in HSPB1-3D to create the HSPB1-3D-GxG construct.

All proteins were expressed in BL21(DE3) or Rosetta 2 (DE3) *E. coli* cells and induced with 1 mM IPTG. HSPB1 constructs were induced at 37 °C for 4 h. They were purified by Ni-affinity chromatography (Nuvia IMAC, Bio-Rad) in buffer 20 mM K_2_HPO_4_/KH_2_PO_4_ pH 7.5, 500 mM NaCl and eluted in the same buffer with 500 mM imidazole followed by a size exclusion chromatography (SEC) using a HiLoad 16/60 Superdex200 (GE Healthcare). DJB1 was expressed at 37 °C for 2 h and purified by Ni-affinity as described for HSPB1 and ion exchange chromatography on a Hi-Trap Q HP column (GE Healthcare), eluted in 20 mM K_2_HPO_4_/KH_2_PO_4_ pH 7.5 with a linear gradient from 50 to 1000 mM NaCl. HSP110 was induced overnight at 20 ℃ and purified through a Ni-affinity chromatography followed by SEC as described for HSPB1.

The His-tags were removed from HSP70, DJA2 and HSPB1 constructs using purified His-tagged tobacco etch virus (TEV) protease and from DJB1 using thrombin protease. The His-SUMO-tag was cleaved from HSP110 by incubation with BdSUMO protease, SENP1^109^. Firefly luciferase and LDH from rabbit muscle were from Sigma. All proteins were dialyzed into reaction buffer (100 mM KOAc, 20 mM HEPES–KOH pH 7.5 and 5 mM MgOAc_2_), except for DJA2 which was stored in buffer containing 500 mM NaCl, 20 mM HEPES–KOH pH 7.5 and 5 mM MgOAc_2_. All purifications were at 4 ℃; proteins purity was analyzed on SDS-PAGE and concentrations were determined by absorbance at 280 nm.

### Aggregation analysis

To assess substrates aggregation, 2 μM luciferase or LDH were incubated at 45 °C or 55 °C, respectively, for 30 min in the presence of 20 μM HSPB1, HSPB1-3D or HSPB1-3D-GxG, in reaction buffer containing 1 mM DTT. At time points, samples were transferred to a quartz cuvette, and optical density at 320 nm due to light scattering was measured on a DU730 UV–Vis spectrophotometer (Beckman Coulter). Control reactions contained HSPB1 or the mutants alone.

### Dynamic light scattering (DLS)

DLS of 20 μM HSPB1 or the mutants in reaction buffer was measured for at least 30 s with 10 repetitions using a quartz cuvette at 8 ℃ in a Zetasizer Nano ZS ZEN3600 (Malvern). Data acquirement and processing were done using the software Zetasizer Nano (Malvern). For aggregates, 2 μM luciferase or LDH in the absence or presence of 20 μM HSPB1 or its mutants were incubated at 45 °C or 55 °C, respectively, for 15 min in reaction buffer, and then measured.

### Cross-linking and immunoblots

For the cross-linking reactions, 2 μM luciferase or LDH were incubated at 4 °C for controls or heat shocked at 45 °C or 55 °C, respectively, in the absence or presence of HSPB1-3D or HSPB1-3D-GxG in reaction buffer. The aggregates were then incubated at room temperature for 5 min with varying concentrations of disuccinimidyl suberate (DSS). Reactions were stopped with 30 mM Tris–HCl pH 7.5 and analyzed by immunoblots with antibodies specific for luciferase (L0159, Sigma), LDH (168–10,648, RayBiotech) and HSPB1 (5D12-A12, StressMarq Biosciences). Chemiluminescence detection was with ECL reagent (GE Healthcare), images were acquired in a ChemiDoc MP imaging system (Bio-Rad) and quantified using the Image Lab software (Bio-Rad) and ImageJ 1.46r (NIH).

### Negative staining electron microscopy

Aggregates in the presence of HSPB1 constructs were prepared through incubation of 2 μM luciferase or LDH and 20 μM HSPB1 or HSPB1-3D for 15 min at 45 °C or 55 °C, respectively. Luciferase alone at 2 µM resulted in very low particle concentration on the grids, so preHS and substrate alone samples were prepared also using 20 µM luciferase or LDH and incubation occurred as described. For all samples, 5 μl of protein solution were applied to freshly glow-discharged carbon-coated copper grids. After a 1 min incubation, the sample was blotted using filter paper, washed twice with ddH_2_O and stained with 0.5% uranyl-acetate for 1 min. Micrographs were collected on a FEI Technai G2 spirit TEM operating at 120 kV equipped with a Gatan Ultrascan 895 CCD.

### Disaggregation and refolding assays

Aggregates were prepared as above by incubating 2 μM luciferase or LDH at 45 °C or 55 °C, respectively, for 15 min in the absence or presence of 20 μM HSPB1 or its mutants in reaction buffer containing 1 mM DTT. Next, samples were diluted 100-fold for luciferase or 25-fold for LDH into reaction buffer, containing 2 μM HSP70, 0.5 μM HSP110, 2 μM DJA2, 1 μM DJB1, 2 mM ATP, and 40 mM NaCl, and incubated at 30 °C for 3 h. Luciferase enzymatic activity was determined at different time points by the addition of luciferase reagent (Promega) to 2 µL of disaggregation reaction. Luminescence was measured in a SIRIUS Tube Luminometer (Berthold). For LDH activity, at different time points, reactions were first stopped with 10 mM EDTA and diluted fivefold into solution containing 2 mM NADH and 2 mM pyruvate. NADH absorbance at 340 nm was monitored for 20 min at 37 °C in a Synergy Mx microplate reader (BioTek) and converted into amount of NADH through a calibration curve. LDH activity was obtained by calculating the slopes of the NADH consumption curves. Native luciferase and LDH activities were measured and set to maximum activity (100%) for all experiments.

Luciferase refolding assays were conducted as published^82,86^; modifications were made for LDH refolding. Briefly, 2 μM luciferase or LDH was denatured in reaction buffer containing 6 M guanidinium-Cl and 1 mM DTT for 10 min at room temperature, then diluted 100-fold for luciferase or 25-fold for LDH into reaction buffer with the addition of chaperones, 2 mM ATP, and 40 mM NaCl, and incubated at 30 °C for 60 min. Luciferase and LDH activities were measured at time points as described. The concentrations of chaperones were the same as for disaggregation experiments.

### Size exclusion chromatography (SEC)

Proteins were separated on a Superose 12 10/300 GL column (GE Healthcare) in reaction buffer at 4 °C, and absorbance at 280 nm was monitored. To analyze aggregates and solubilization, aggregates were formed as above, and analyzed immediately or after 3 h solubilization, or after mock reactions without chaperones. Fractions of 1 mL size were collected from 6 to 18 mL elution volume and proteins were precipitated by addition of 15% w/v trichloroacetic acid and 0.1 mg/mL ovalbumin or 0.1 mg/mL BSA followed by centrifugation at 20,000×g for 30 min at 4 °C, the pellets were washed with acetone, centrifuged again at 20,000×g for 15 min at 4 °C and resuspended in SDS-PAGE sample buffer adjusted above pH 7 with Tris base. The elution pattern of the proteins was then evaluated by immunoblots as above.

## Supplementary Information

Supplementary Information.

## Data Availability

This study includes no data deposited in external repositories.
