# Supplementary material for: The chaperone HSPB1 prepares protein aggregates for resolubilization by HSP70
Source: Sci Rep. 2021 Aug 24;11:17139. doi: 10.1038/s41598-021-96518-x (PMC8384840; doi:10.1038/s41598-021-96518-x)

The chaperone HSPB1 prepares protein aggregates for resolubilization by HSP70

Conrado C. Gonçalves[1], Itai Sharon[2], T. Martin Schmeing[2], Carlos H. I. Ramos[3] and Jason C. Young[1]*.

[1] McGill University, Department of Biochemistry, 3655 Promenade Sir William Osler, Room 900, Montreal, QC, Canada H3G 1Y6

[2] McGill University, Department of Biochemistry, 3649 Promenade Sir William Osler, Room 457, Montreal, QC, Canada H3G 0B1

[3] University of Campinas (UNICAMP), Institute of Chemistry, Campinas, SP, 13083-970, Brazil

* Correspondence to: jason.young2@mcgill.ca

Supplementary Figure Legends

Figure S1. Intermolecular luciferase-luciferase and LDH-LDH contacts form in co-aggregates with HSPB1-3D. (a) Cross-linking analysis of co-aggregates composed of 2 µM luciferase and 20 µM HSPB1-3D. Samples were incubated at 4 °C or under heat-shock (HS) at 45 °C, in the absence or presence of HSPB1-3D and submitted to cross-linking reactions using the indicated concentrations of DSS and then analyzed by immunoblot (IB). Monomeric species that did not cross-link, as well as cross-linked aggregates or oligomers are indicated. **(b, c)** Cross-linking analysis of co-aggregates composed of 2 µM LDH and 20 µM **(b)** HSPB1-3D or **(c)** HSPB1-3D-GxG. Reactions were conducted as in a, except under heat shock (HS) at 55 °C.

Figure S2. HSPB1 remains mainly as an heterogenous oligomer during disaggregation. **(a-c)** The elution patterns of **(a)** HSPB1 before the heat shock (preHS) or in the disaggregation reactions with **(b)** luciferase or **(c)** LDH were assessed by immunoblot (IB). **(d, e)** Quantification of HSPB1 in the reactions with **(d)** luciferase and **(e)** LDH from b and c are shown as percentage of the total amount of immunoblot signal. Error bars show standard deviations, n ≥ 3.

Figure S3. Heat shocked HSPB1-3D forms oligomeric species in the absence of substrate. (a) Elution patterns of HSPB1 in disaggregation reactions in the absence of substrate. Reactions were performed as in Fig. 4f, except with heat shock at 55 °C. The elution patterns before or after heat shock were assessed at the start (0 h) and end (3 h) of disaggregation reactions with or without

chaperones by SEC and immunoblot (IB). **(b)** Quantification of HSPB1 from a in eluted fractions are shown as percentage of the total amount of immunoblot signal. **(c)** Elution patterns of HSPB1-3D in disaggregation reactions in the absence of substrate as in a. **(d)** Quantification of HSPB1-3D from c in eluted fractions are shown as percentage of the total amount of immunoblot signal. Error bars show standard deviations, n ≥ 3.

Figure S4. Luciferase disaggregation with excess HSPB1-3D. (a) Disaggregation of luciferase aggregates with 20 or 80 µM HSPB1-3D. The aggregates were formed at 45 °C for 15 min and transferred to disaggregation reactions containing buffer (- Chap) or the chaperone disaggregation machinery (+ Chap) and ATP. Aggregate resolubilization at the start (0 h) and end (3h) of the disaggregation reactions was analyzed by SEC and immunoblot (IB). **(b-d)** Quantification of **(b)** luciferase and HSPB1-3D at **(c)** 20 µM and **(d)** 80 µM from a in eluted fractions are shown as percentage of the total amount of immunoblot signal. Error bars show standard deviations, n ≥ 3.

Figure S5. Luciferase refolding is dependent on the HSP70-DJA2 chaperone system while LDH refolding is spontaneous. Guanidine-denatured **(a, b)** luciferase or **(c, d)** LDH was diluted into refolding reactions containing HSP70 and the indicated combinations of co-chaperones. **(a, c)** Kinetics of substrate refolding was determined by measuring substrate enzymatic activity recovery over time. **(c)** Luciferase and **(d)** LDH reactivation as in a and c, after 60 min of refolding reaction with different combinations of chaperones. Native substrate activity was set as maximum activity (100 %). Reactions in the absence of chaperones or HSP70 were used as controls. Error bars show standard deviations, n ≥ 3.

Figure S6. Co-aggregation with HSPB1-3D-GxG affects LDH and luciferase aggregates differently. (a, b) Aggregation of **(a)** luciferase and **(b)** LDH was assessed as in Fig. 1a, b in solutions containing 2 µM substrate with or without 20 µM HSPB1-3D-GxG. Error bars show standard deviations, n = 3. **(c, d)** The size distribution of **(c)** luciferase and **(d)** LDH aggregates formed with HSPB1-3D-GxG was estimated by DLS and compared to the size of HSPB1-3D-GxG alone and substrate alone aggregates.

Figure S7. Heat shocked HSPB1-3D-GxG remains as a dimer and does not contribute to luciferase resolubilization. (a) Elution pattern of HSPB1-3D-GxG in disaggregation reactions in the absence of substrate. Reactions were performed as in Fig. 4f, except with heat shock at 55 °C. The elution patterns before or after heat shock were assessed at the start (0 h) and end (3 h) of disaggregation reactions with or without chaperones by SEC and immunoblot (IB). **(b)** Quantification of HSPB1-3D-GxG from a in eluted fractions are shown as percentage of the total amount of immunoblot signal. Error bars show standard deviations, n ≥ 3. **(c)** Disaggregation reactions with luciferase were performed as in Fig. 4 except with HSPB1-3D-GxG. Aggregate solubilization at the start (0 h) and end (3h) of the disaggregation reactions was assessed by SEC and immunoblot (IB).

Figure S8. Original Western blots used in figures.

Figure S1

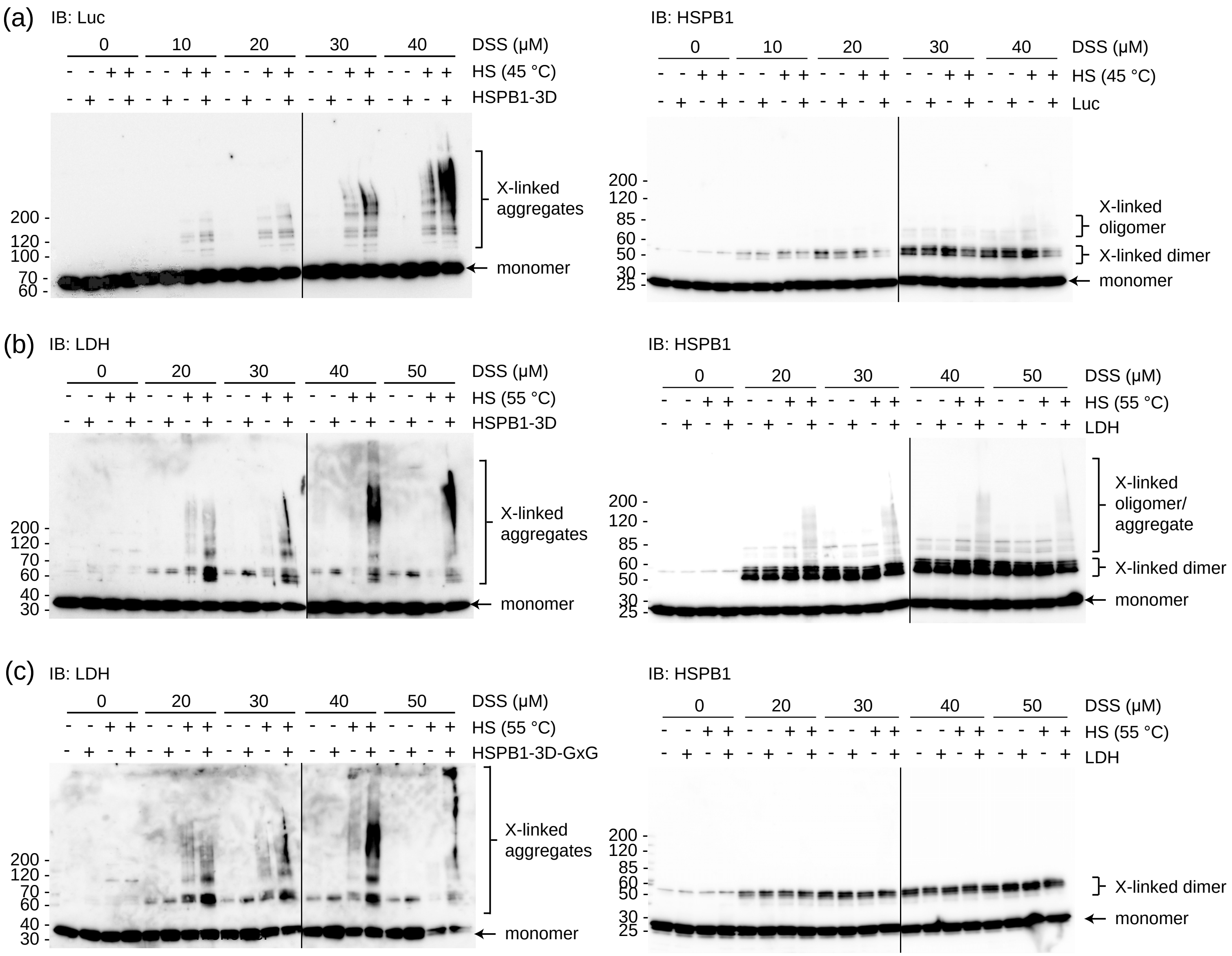
(a)
IB: Luc
DSS (μM)
0
10
20
30
40
HS (45 °C)
HSPB1-3D
200 -
120 -
100 -
70 -
60 -
X-linked aggregates
monomer
IB: HSPB1
Luc
X-linked oligomer
X-linked dimer
85 -
50 -
30 -
25 -
(b)
IB: LDH
HS (55 °C)
X-linked oligomer/ aggregate
40 -
(c)
HSPB1-3D-GxG
LDH

Figure S2

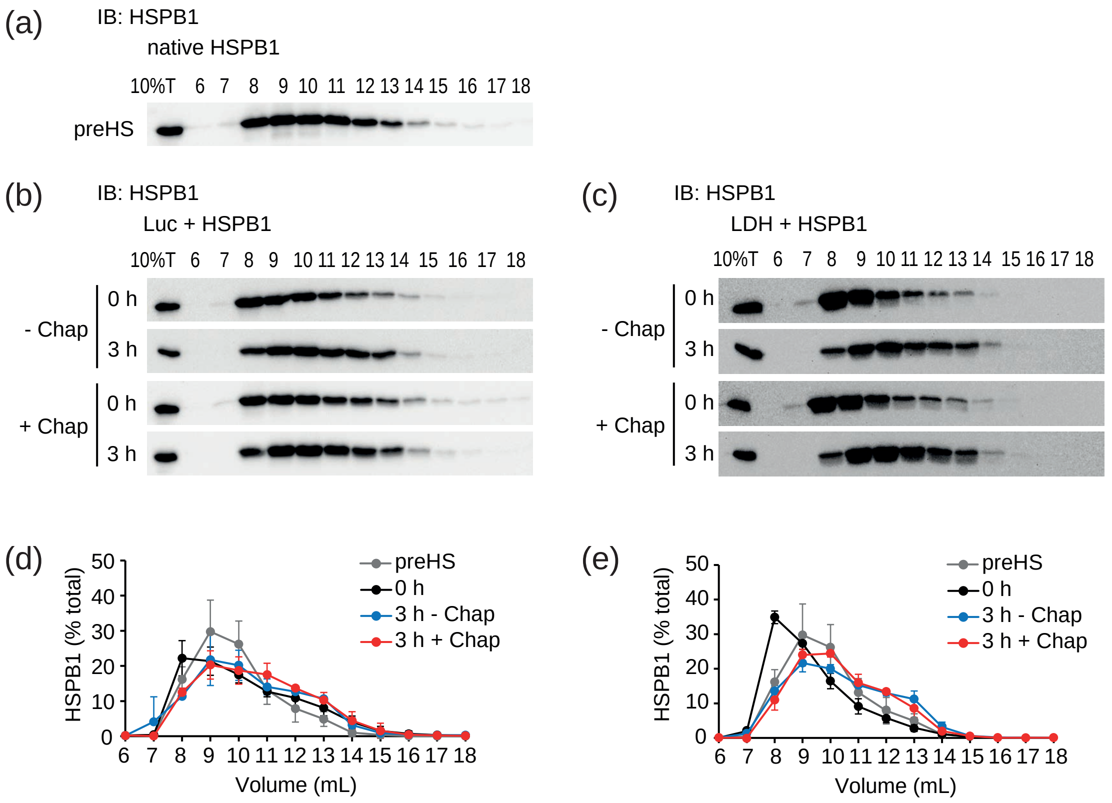

(a)
IB: HSPB1
native HSPB1
10%T 6 7 8 9 10 11 12 13 14 15 16 17 18
preHS
(b)
IB: HSPB1
Luc + HSPB1
10%T 6 7 8 9 10 11 12 13 14 15 16 17 18
- Chap
0 h
3 h
+ Chap
0 h
3 h
(c)
IB: HSPB1
LDH + HSPB1
10%T 6 7 8 9 10 11 12 13 14 15 16 17 18
- Chap
0 h
3 h
+ Chap
0 h
3 h
(d)
HSPB1 (% total)
0
10
20
30
40
50
6 7 8 9 10 11 12 13 14 15 16 17 18
Volume (mL)
preHS
0 h
3 h - Chap
3 h + Chap
(e)
HSPB1 (% total)
0
10
20
30
40
50
6 7 8 9 10 11 12 13 14 15 16 17 18
Volume (mL)
preHS
0 h
3 h - Chap
3 h + Chap

Figure S3

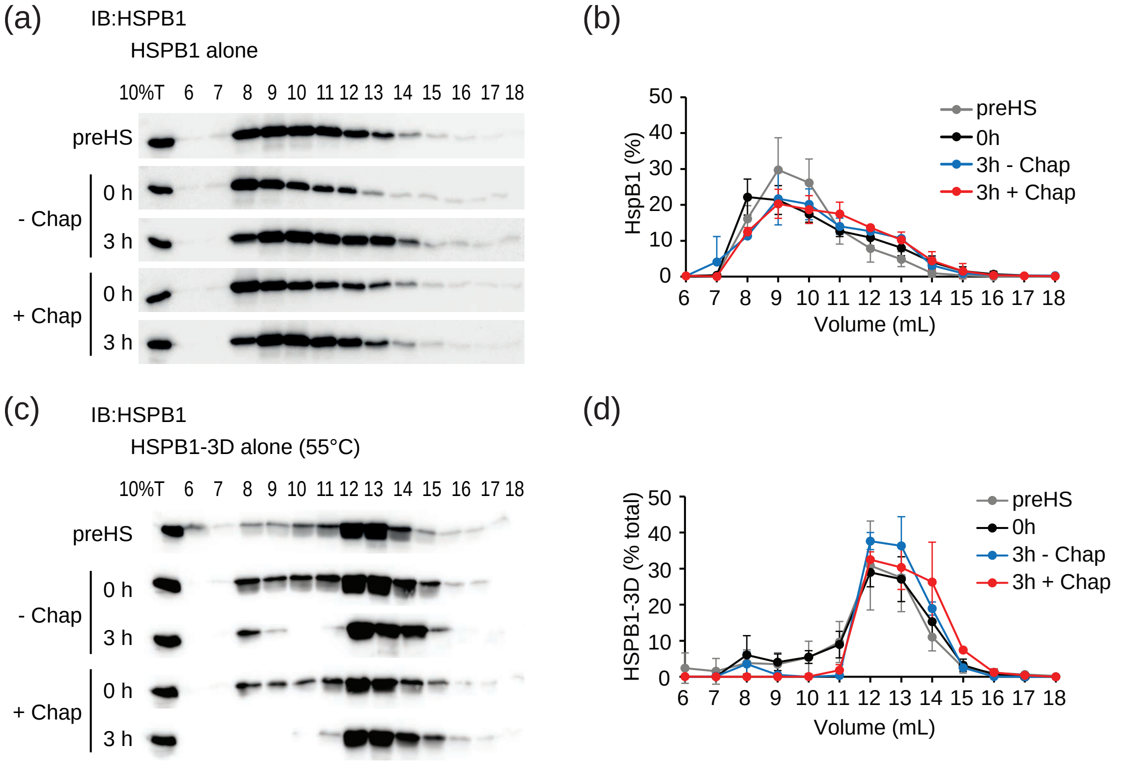
(a)
IB:HSPB1
HSPB1 alone
10%T 6 7 8 9 10 11 12 13 14 15 16 17 18
preHS
- Chap
0 h
3 h
+ Chap
0 h
3 h
(b)
HspB1 (%)
Volume (mL)
preHS
0h
3h - Chap
3h + Chap
(c)
IB:HSPB1
HSPB1-3D alone (55°C)
10%T 6 7 8 9 10 11 12 13 14 15 16 17 18
preHS
- Chap
0 h
3 h
+ Chap
0 h
3 h
(d)
HSPB1-3D (% total)
Volume (mL)
preHS
0h
3h - Chap
3h + Chap

Figure S4

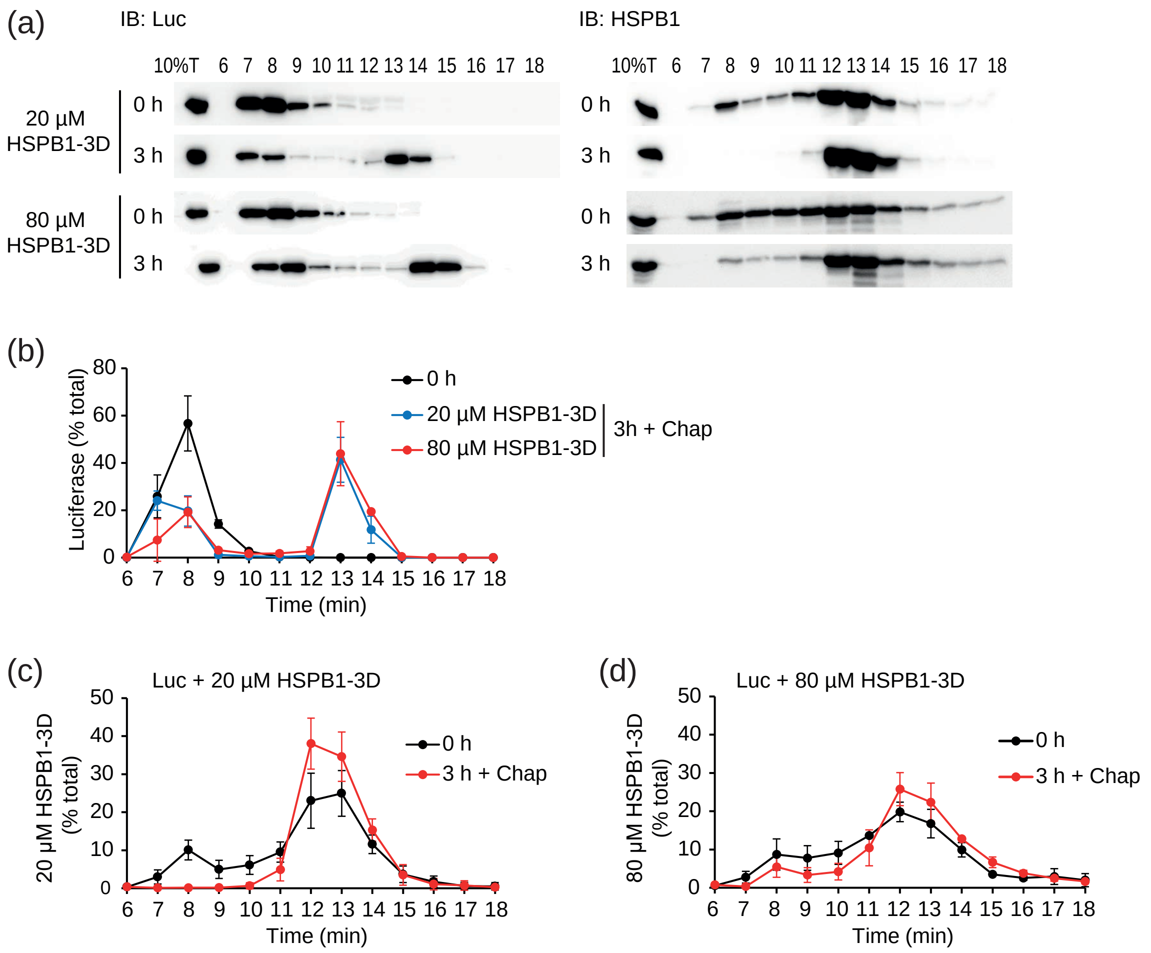
(a)
IB: Luc
IB: HSPB1
10%T 6 7 8 9 10 11 12 13 14 15 16 17 18
20 µM HSPB1-3D
80 µM HSPB1-3D
0 h
3 h
(b)
Luciferase (% total)
Time (min)
0 h
20 µM HSPB1-3D
80 µM HSPB1-3D
3h + Chap
(c)
Luc + 20 µM HSPB1-3D
20 µM HSPB1-3D (% total)
0 h
3 h + Chap
Time (min)
(d)
Luc + 80 µM HSPB1-3D
80 µM HSPB1-3D (% total)
0 h
3 h + Chap
Time (min)

Figure S5

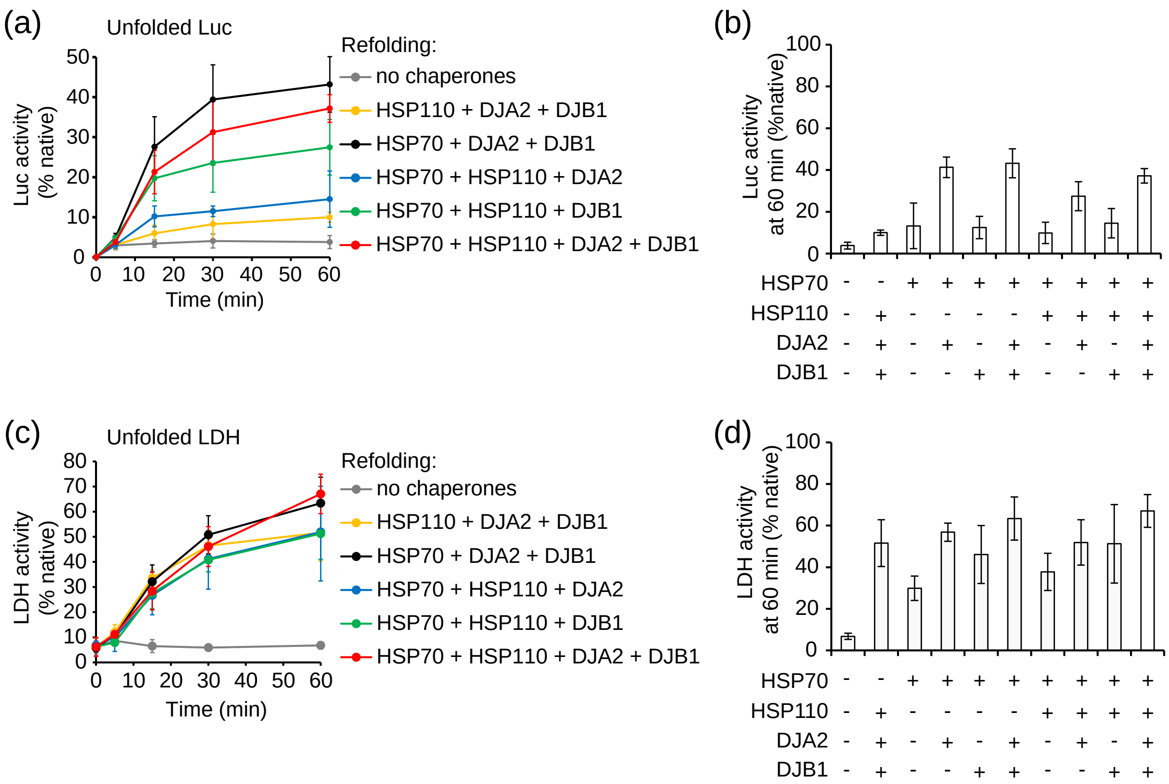

(a)
Unfolded Luc
Luc activity (% native)
Time (min)
Refolding:
no chaperones
HSP110 + DJA2 + DJB1
HSP70 + DJA2 + DJB1
HSP70 + HSP110 + DJA2
HSP70 + HSP110 + DJB1
HSP70 + HSP110 + DJA2 + DJB1
(b)
Luc activity at 60 min (%native)
HSP70 - - + + + + + + + +
HSP110 - + - - - - + + + +
DJA2 - + - + - + - + - +
DJB1 - + - - + + - - + +
(c)
Unfolded LDH
LDH activity (% native)
Time (min)
Refolding:
no chaperones
HSP110 + DJA2 + DJB1
HSP70 + DJA2 + DJB1
HSP70 + HSP110 + DJA2
HSP70 + HSP110 + DJB1
HSP70 + HSP110 + DJA2 + DJB1
(d)
LDH activity at 60 min (% native)
HSP70 - - + + + + + + + +
HSP110 - + - - - - + + + +
DJA2 - + - + - + - + - +
DJB1 - + - - + + - - + +

Figure S6

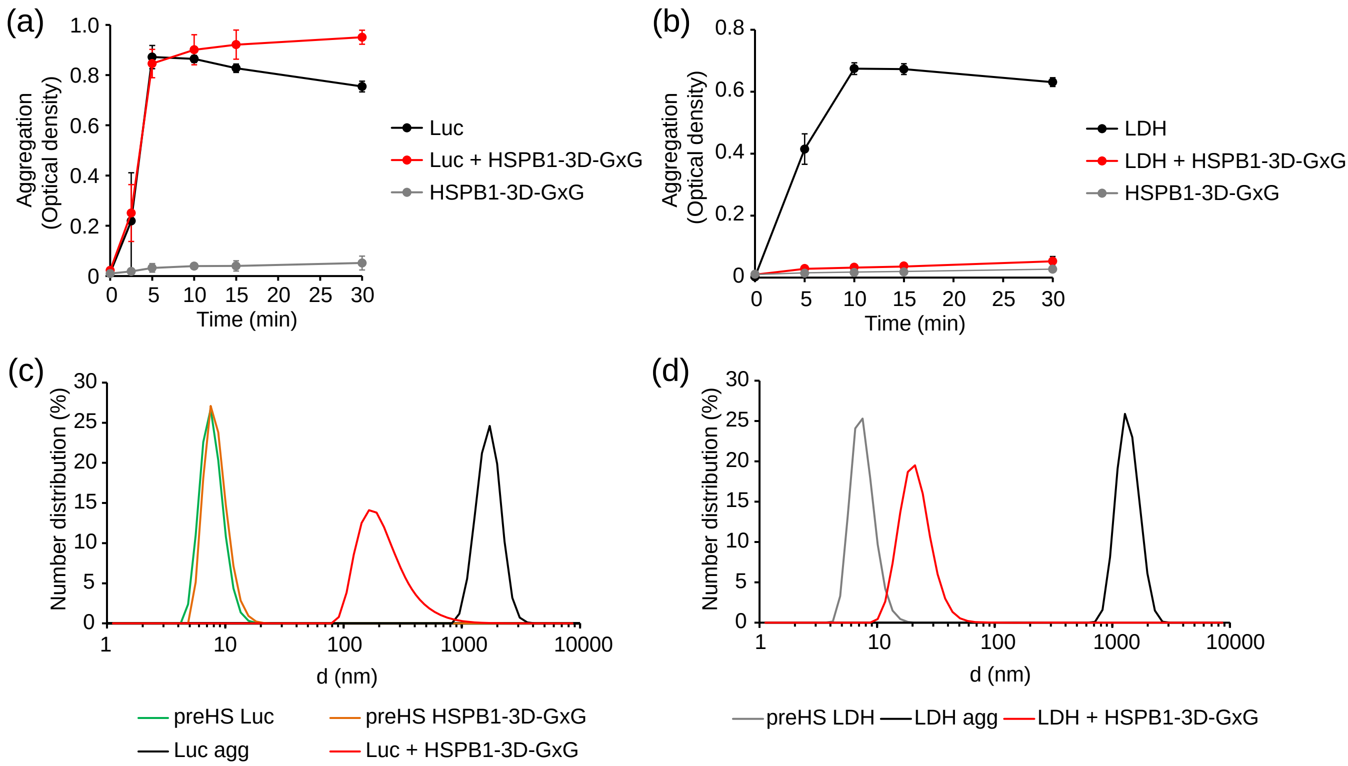
(a)
Aggregation
(Optical density)
Time (min)
Luc
Luc + HSPB1-3D-GxG
HSPB1-3D-GxG
(b)
LDH
LDH + HSPB1-3D-GxG
HSPB1-3D-GxG
(c)
Number distribution (%)
d (nm)
preHS Luc
preHS HSPB1-3D-GxG
Luc agg
Luc + HSPB1-3D-GxG
(d)
preHS LDH
LDH agg
LDH + HSPB1-3D-GxG

Figure S7

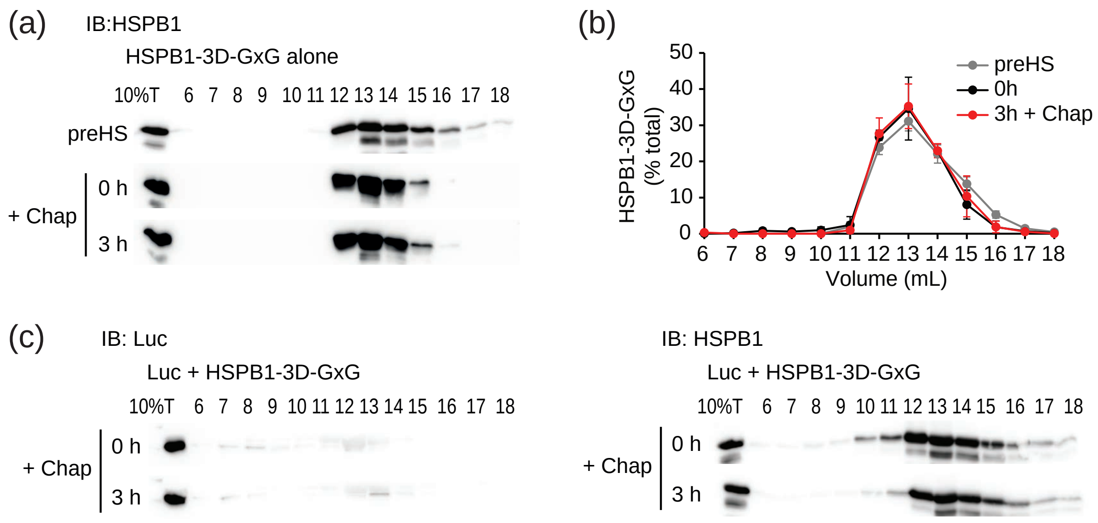

(a)
IB:HSPB1
HSPB1-3D-GxG alone
10%T 6 7 8 9 10 11 12 13 14 15 16 17 18
preHS
+ Chap
0 h
3 h
(b)
HSPB1-3D-GxG (% total)
50
40
30
20
10
0
6 7 8 9 10 11 12 13 14 15 16 17 18
Volume (mL)
preHS
0h
3h + Chap
(c)
IB: Luc
Luc + HSPB1-3D-GxG
10%T 6 7 8 9 10 11 12 13 14 15 16 17 18
+ Chap
0 h
3 h
IB: HSPB1
Luc + HSPB1-3D-GxG
10%T 6 7 8 9 10 11 12 13 14 15 16 17 18
+ Chap
0 h
3 h

Figure S8

Original Western blots

Figure 3a

IB: Luc

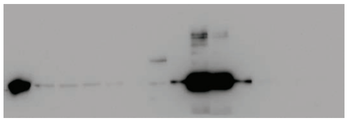

Figure 3b

IB: LDH

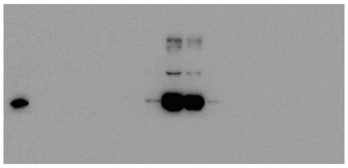

Figure 3c

IB: Luc

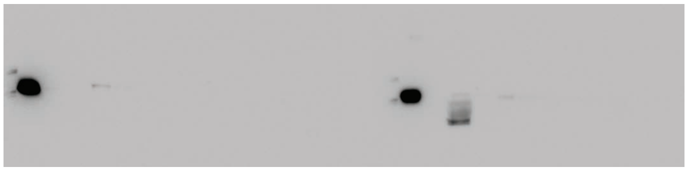

IB: Luc

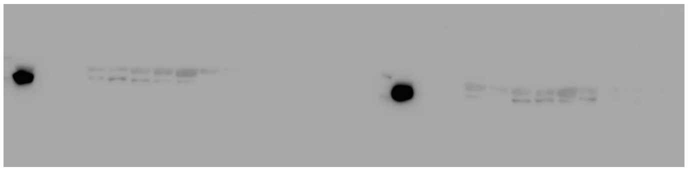

Figure 3d

IB: LDH

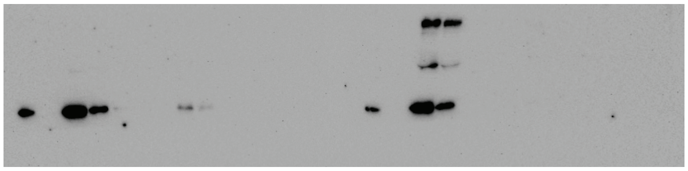

IB: LDH

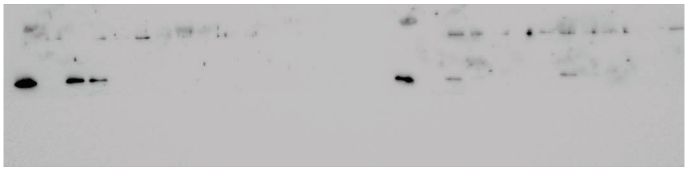

Figure 3e

IB: Luc

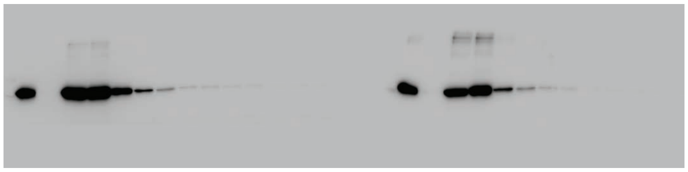

IB: Luc

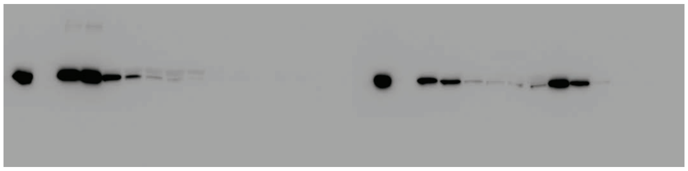

Figure 3f

IB: LDH

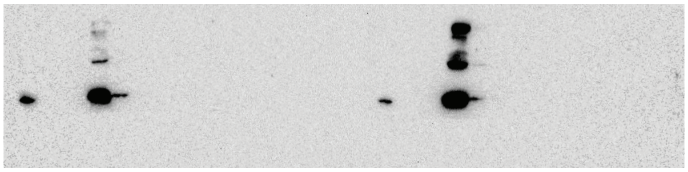

IB: LDH

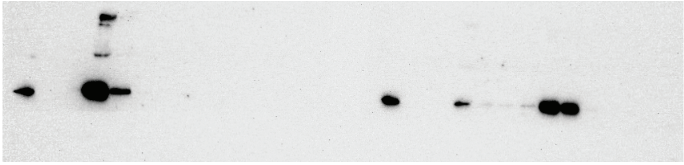

Figure 4a

IB: HSPB1

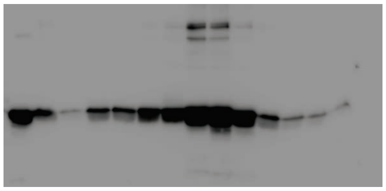

Figure 4b

IB: HSPB1

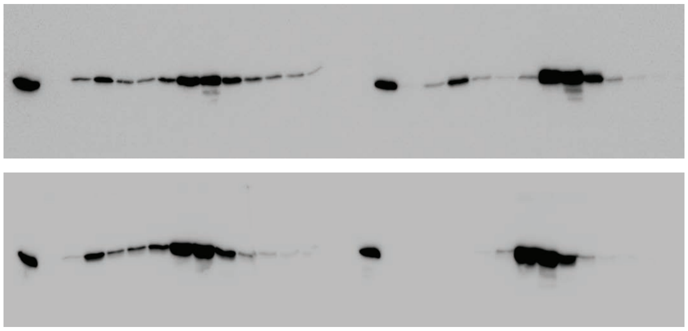

Figure S8 *(cont.)*

Original Western blots

Figure 4c

IB: HSPB1

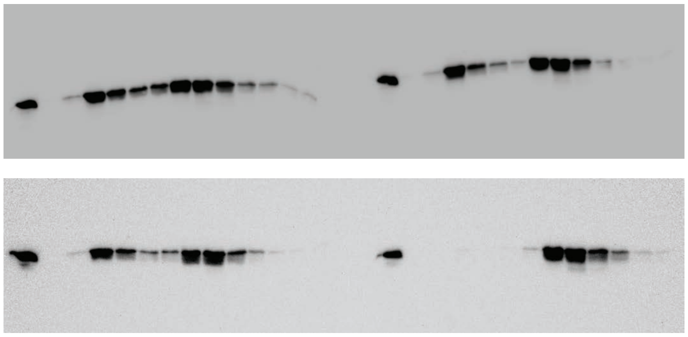

Figure 4f

IB: HSPB1

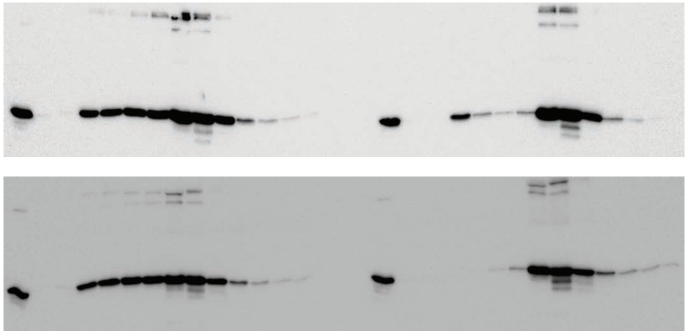

Figure 6c

IB: LDH

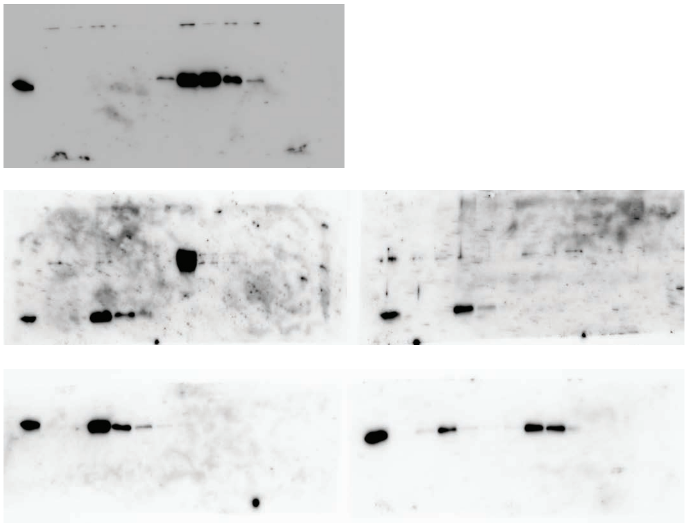

Figure 6d

IB: HSPB1

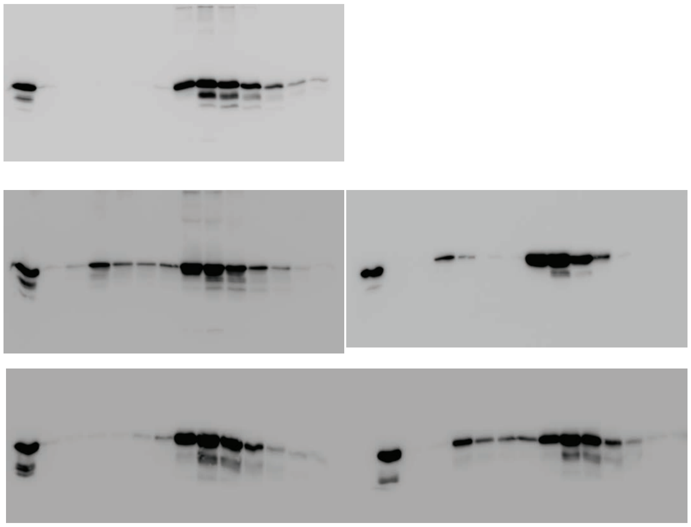

Figure S8 *(cont.)*

Original Western blots

Figure S1a

IB: Luc

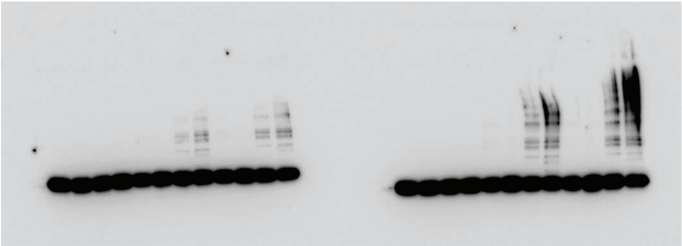

IB: HSPB1

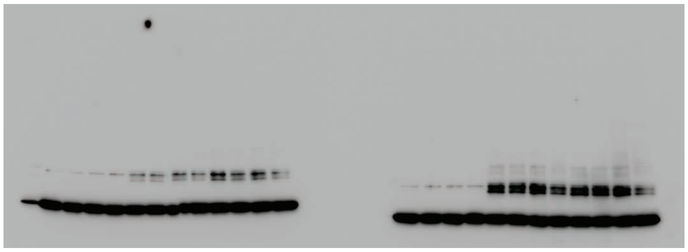

Figure S1b

IB: LDH

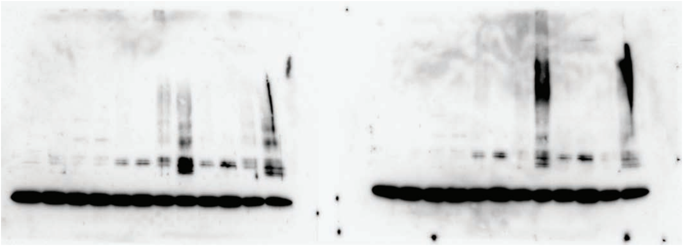

IB: HSPB1

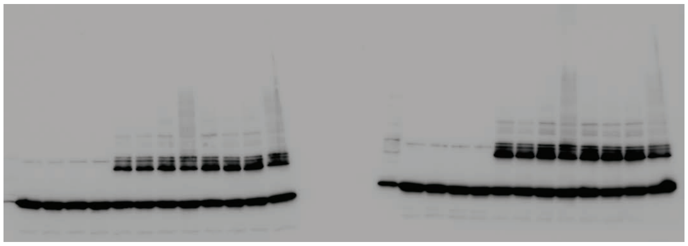

Figure S1c

IB: LDH

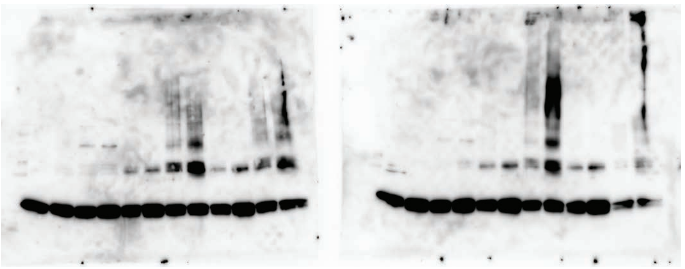

IB: HSPB1

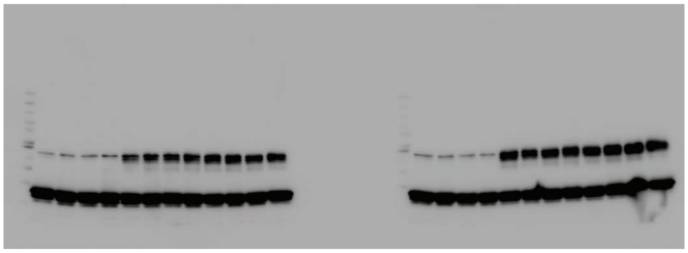

Figure S2a

IB: HSPB1

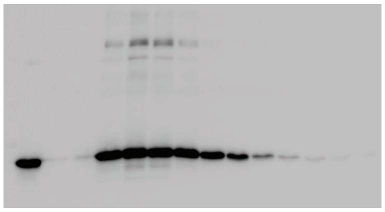

Figure S8 *(cont.)*

Original Western blots

Figure S2b

IB: HSPB1

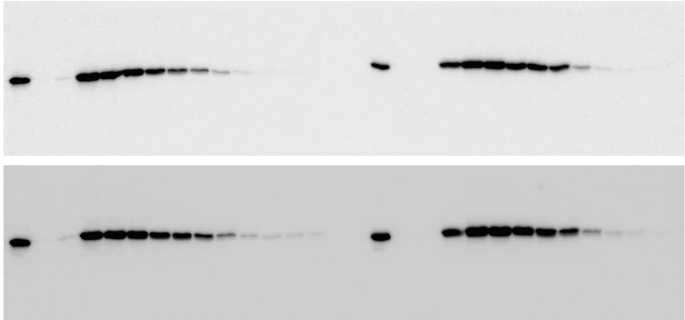

Figure S2c

IB: HSPB1

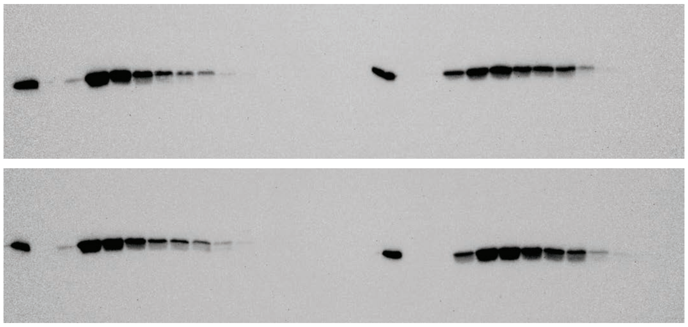

Figure S3a

IB: HSPB1

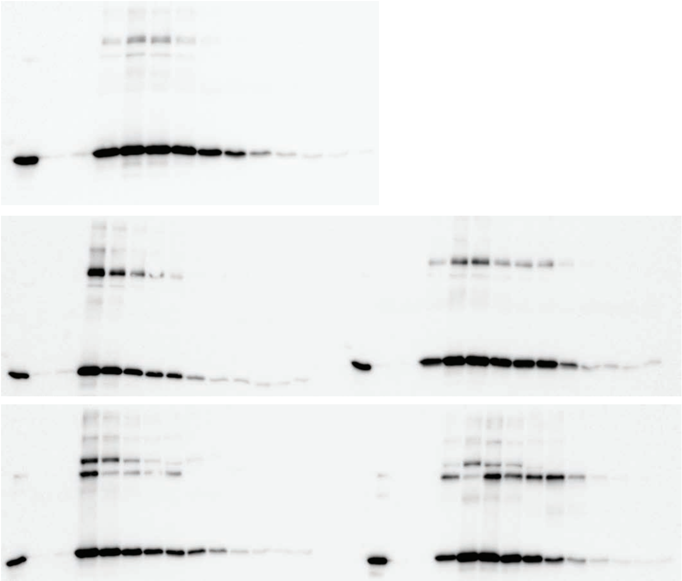

Figure S3c

IB: HSPB1

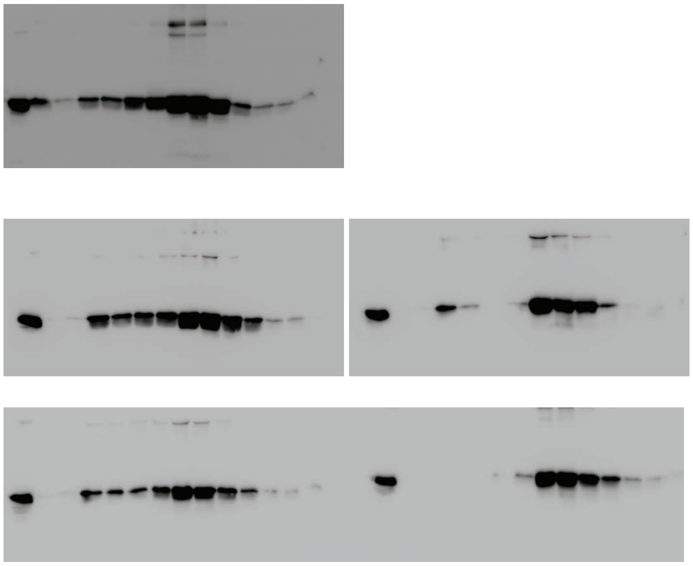

Figure S8 *(cont.)*

Original Western blots

Figure S4a

IB: Luc

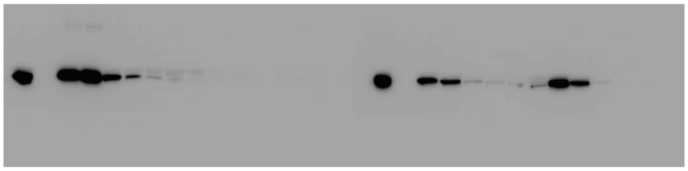

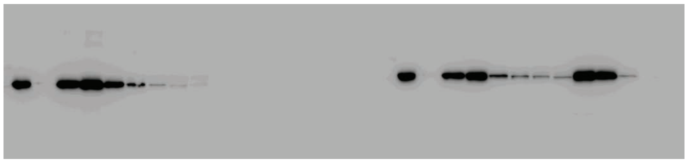

IB: HSPB1

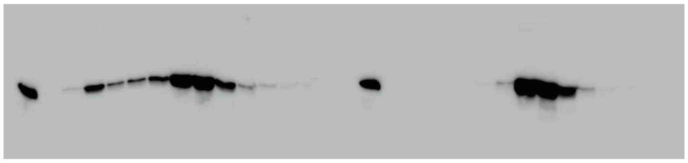

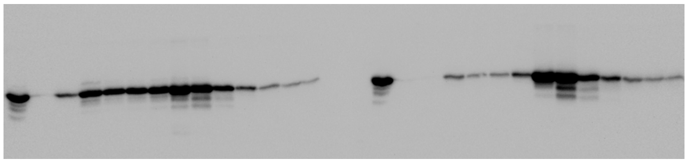

Figure S7a

IB: HSPB1

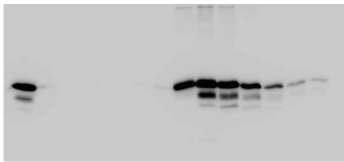

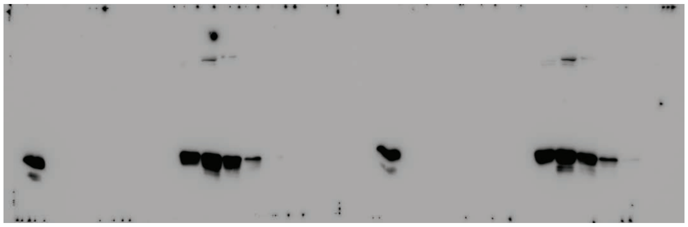

Figure S7c

IB: Luc

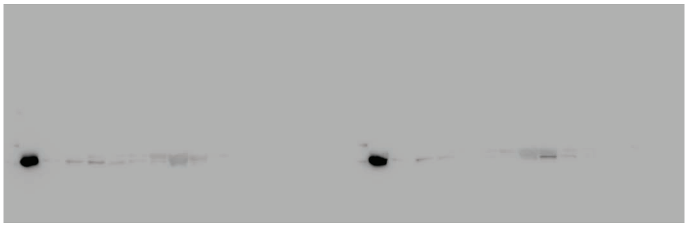

IB: HSPB1

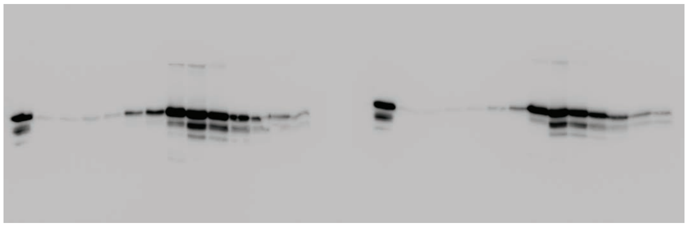

Supplement: Supplementary file 1 — Supplementary Information. [file 41598_2021_96518_MOESM1_ESM.pdf]
